# Brainstem ADCYAP1^+^ neurons control multiple aspects of sickness behaviour

**DOI:** 10.1038/s41586-022-05161-7

**Published:** 2022-09-07

**Authors:** Anoj Ilanges, Rani Shiao, Jordan Shaked, Ji-Dung Luo, Xiaofei Yu, Jeffrey M. Friedman

**Affiliations:** 1 Laboratory of Molecular Genetics, The Rockefeller University, New York, NY USA; 2 Bioinformatics Resource Center, The Rockefeller University, New York, NY USA; 3 State Key Laboratory of Genetic Engineering, School of Life Sciences, Fudan University, Shanghai, China; 4 Howard Hughes Medical Institute, The Rockefeller University, New York, NY USA

**Keywords:** Neuroimmunology, Feeding behaviour, Neural circuits, Neuroimmunology

## Abstract

Infections induce a set of pleiotropic responses in animals, including anorexia, adipsia, lethargy and changes in temperature, collectively termed sickness behaviours^1^. Although these responses have been shown to be adaptive, the underlying neural mechanisms have not been elucidated^2–4^. Here we use of a set of unbiased methodologies to show that a specific subpopulation of neurons in the brainstem can control the diverse responses to a bacterial endotoxin (lipopolysaccharide (LPS)) that potently induces sickness behaviour. Whole-brain activity mapping revealed that subsets of neurons in the nucleus of the solitary tract (NTS) and the area postrema (AP) acutely express FOS after LPS treatment, and we found that subsequent reactivation of these specific neurons in FOS^2A-iCreERT2^ (also known as TRAP2) mice replicates the behavioural and thermal component of sickness. In addition, inhibition of LPS-activated neurons diminished all of the behavioural responses to LPS. Single-nucleus RNA sequencing of the NTS–AP was used to identify LPS-activated neural populations, and we found that activation of ADCYAP1^+^ neurons in the NTS–AP fully recapitulates the responses elicited by LPS. Furthermore, inhibition of these neurons significantly diminished the anorexia, adipsia and locomotor cessation seen after LPS injection. Together these studies map the pleiotropic effects of LPS to a neural population that is both necessary and sufficient for canonical elements of the sickness response, thus establishing a critical link between the brain and the response to infection.

## Abstract (web summary)

A studying using a set of unbiased methodologies shows that a specific subpopulation of neurons in the brainstem can regulate the diverse responses to a bacterial endotoxin that induces sickness behaviours.

## Main

Infections are an omnipresent threat to all species, and effective adaptive responses are essential for survival. Mechanisms that enable animals to survive an infection are thus under intense selective pressure^5^. The response to infectious agents is not limited to immune cells but also includes a coordinated set of responses collectively known as sickness behaviour^1–3^. Thus, animals infected with pathogens, or their components such as LPS, develop anorexia, adipsia and lethargy, and also show altered thermoregulation. Moreover, forced feeding of animals^4,6,7^ or changing the thermal environment^8^ after bacterial infection or treatment with LPS increases mortality, showing that these responses are part of a coordinated adaptive response.

However, although bacteria, endotoxins and the cytokines they induce can elicit aspects of the sickness response^9–12^, the neural substrate for the behavioural and autonomic effects of infection has not been elucidated^13,14^, nor is it clear whether one or more neuronal populations are required for different aspects of the response. Here we report that multiple different responses to LPS can be mapped to a core population in the sensory dorsal vagal complex of the brainstem expressing the neuropeptide ADCYAP1. These studies thus identify a critical node linking the immune response to a series of adaptive responses coordinated by the brain.

## LPS induces coordinated sickness behaviours

**Extended Data Fig. 1:**
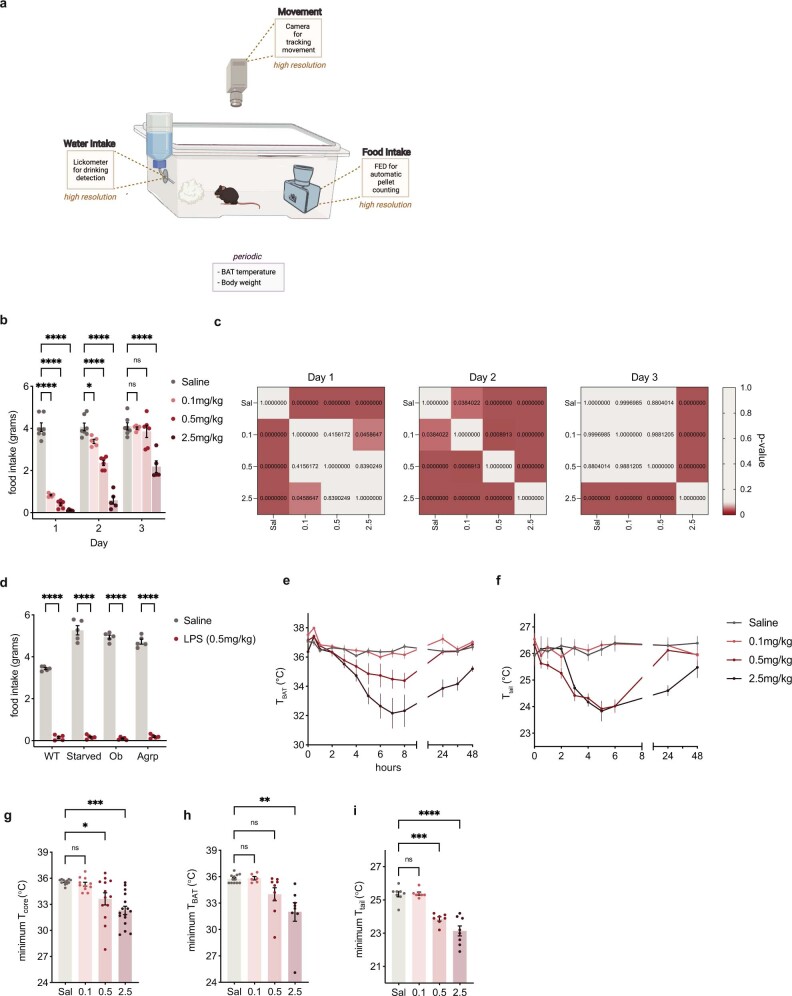
High-resolution phenotyping of sickness behaviors. **a**, Schematic of phenotyping cages used for measurement of food intake, water intake, and movement. **b**, Daily food intake for saline (grey) and LPS doses of 0.1mg/kg (light red), 0.5mg/kg (red), and 2.5mg/kg (dark red). **c**, P-value statistics from RM two-way ANOVA for each day of food intake. **d**, 24 h food intake for different hyperphagic models. WT, wildtype; Starved, wildtype animals starved for 24 h; Ob, leptin deficient animals; AgRP, AgRP-cre animals with stereotactic injection of activating DREADDs in the arcuate region stimulated with CNO. Saline injection (grey) and 0.5mg/kg LPS injection (red) is shown for each animal model. LPS inhibited food intake in all four of these animal models. **e**,**f**, BAT temperature (T_BAT_) (**e**) and tail temperature (T_tail_) (**f**)time course for 48 h after injection of saline (N = 12 and N = 8, grey) or LPS (0.1mg/kg, light red, N = 6 and N = 7; 0.5mg/kg, medium red, N = 9 and N = 7; 2.5mg/kg, dark red, N = 8 and N = 8). **g**-**i**, Minimal recorded core (**g**), BAT (**h**), and tail temperature (**i**) for animals from time course experiments. *P < 0.05, **P < 0.01, ***P < 0.001, ****P < 0.0001 and NS, not significant (P > 0.05). All error bars represent s.e.m. The mouse illustration in **a** was created using BioRender.com. Source data

Sickness behaviour can be induced by bacterial infections or endotoxin treatment, and the qualitative response to each is indistinguishable^15^. We studied the sickness responses using LPS, which yields a more consistent response than does bacterial infection. The use of LPS also enables one to control for variability in individual animal responses to an infection while also dissociating host responses from pathogen-induced effects that damage and alter organ function^16^. We thus used LPS to study sickness behaviour and established a dose–response relationship for each of its behavioural effects over time. The lethal dose of LPS is about 10 mg kg^−1^, and we tested the effects of a single intraperitoneal (i.p.) injection of three lower doses of LPS (0.1 mg kg^−1^, 0.5 mg kg^−1^ and 2.5 mg kg^−1^) on each of the components of sickness behaviour using a custom home-cage system that we developed (Extended Data Fig. 1a). This system enables the simultaneous real-time measurement of food intake, water intake and movement in an automated and continuous manner with high resolution. We also periodically monitored core body temperature, brown adipose tissue (BAT) temperature, tail temperature and body weight.

**Fig. 1:**
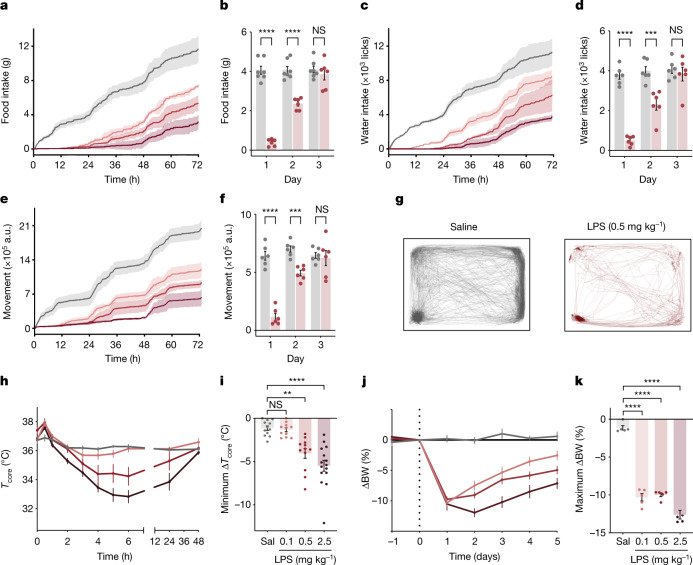
LPS induces coordinated, dose-dependent behavioural and thermal changes. **a**,**c**,**e**, Cumulative food intake (**a**), water intake (**c**) and movement (**e**) for 72 h after injection of saline (grey; *N* = 7) or LPS at 0.1 mg kg^−1^ (light red; *N* = 6), 0.5 mg kg^−1^ (middle red; *N* = 6) or 2.5 mg kg^−1^ (dark red; *N* = 6). Solid line, mean; shaded area, 95% confidence interval. **b**,**d**,**f**, Food intake (**b**), water intake (**d**) and movement (**f**) for each day post injection with saline (grey) or 0.5 mg kg^−1^ LPS (red). For all three parameters, there were significant reductions on the first and second days post LPS injection, but full recovery by the third day (two-way ANOVA first day: food intake *P* < 1 × 10^−7^, water intake *P* < 1.6 × 10^−6^, movement *P* = 3 × 10^−6^). **g**, Movement trace for the first 24 h post saline or LPS (0.5 mg kg^−1^) for a representative mouse. **h**, Time course of the core temperature (*T*_core_) for 48 h after injection of saline (grey; *N* = 12) or LPS at 0.1 mg kg^−1^ (light red; *N* = 11), 0.5 mg kg^−1^ (middle red; *N* = 12) or 2.5 mg kg^−1^ (dark red; *N* = 15). Points, mean; error bars, s.e.m. **i**, Minimum change in *T*_core_ for each dose at 48 h post injection (two-way ANOVA: 0.1 mg kg^−1^ *P* = 1.0, 0.5 mg kg^−1^ *P* = 2.8 × 10^−3^, 2.5 mg kg^−1^ *P* = 4.9 × 10^−5^; colours as in **h**; Sal, saline).  **j**, Percentage change in body weight (BW) from day 0 (baseline) for animals injected with saline or LPS (0.1 mg kg^−1^, *n* = 6; 0.5 mg kg^−1^, *n* = 6; and 2.5 mg kg^−1^, *n* = 6; colours as in **h**) on day 0. **k**, Maximal percentage decrease in body weight following injection (two-way ANOVA: *P* < 1 × 10^−7^ for 0.1 mg kg^−1^, 0.5 mg kg^−1^ and 2.5 mg kg^−1^; colours as in **h**). **P < 0.01, ***P < 0.001, ****P < 0.0001 and NS, not significant (*P* > 0.05). All error bars represent the s.e.m. Source data

All three doses of LPS led to rapid and marked changes of the aforementioned behavioural parameters with a clear dose–response relationship relative to saline controls (Fig. 1). Food and water intake and locomotor activity were significantly decreased within the first 15 min post injection for all three doses (Fig. 1a,c,e). By 24 h, animals treated with LPS (0.5 mg kg^−1^) showed negligible food intake (4.06 ± 0.20 g and 0.39 ± 0.72 g for saline and LPS, respectively; analysis of variance (ANOVA) *P* < 1 × 10^−7^), negligible water intake (3,761.50 ± 184.07 licks and 489.50 ± 93.65 licks for saline and LPS, respectively; ANOVA *P* = 1.6 × 10^−6^) and a near cessation of movement (642,525.24 ± 37,808.63 arbitrary units (a.u.) and 116,816.60 ± 29,619.42 a.u. for saline and LPS, respectively; ANOVA *P* = 3 × 10^−6^; Fig. 1a–g and Extended Data Fig. 1b,c). The time course for induction and recovery for each of these responses was nearly identical, suggesting that these responses are highly coordinated. These responses culminated in dose-dependent body weight loss, with all three doses resulting in a substantial decrease in body weight in the first day and the highest dose causing a further decrease on the second day (Fig. 1j,k). To test whether the behavioural effects observed during sickness behaviour are dominant to homeostatic mechanisms, we tested the response of LPS during three different conditions associated with hyperphagia: starvation, leptin deficiency and chemogenetic (hM3Dq-DREADDs) activation of Arc^AgRP+^ neurons. LPS-induced anorexia was evident in all three models, resulting in nearly identical reductions in food intake to that of wild-type animals treated with LPS, showing that LPS suppresses eating even when the homeostatic drive to eat is activated (Extended Data Fig. 1d).

We also followed the effect of LPS on core temperature. There was a decrease in core temperature at 2 h post LPS injection that was significant only with the highest dose (36.2 ± 0.1 °C and 35.3 ± 0.2 °C for saline and LPS respectively; ANOVA *P* = 0.003; Fig. 1h,i and Extended Data Fig. 1g). The core temperature for the intermediate dose trended downward at 3 h post LPS injection but did not elicit a significant decrease until 5 h after injection (36.3 ± 0.1 °C and 34.4 ± 0.6 °C for saline and LPS, respectively; ANOVA *P* = 0.0494). The lowest LPS dose did not cause a significant change in core temperature. We next assayed BAT temperature and tail temperature. The BAT serves as a main source of heat production in mice, whereas increased blood flow to the tail can promote dissipation of heat. As for the effects of LPS on core temperature, the BAT temperature increased in the first 30 min following LPS treatment, with a significant change observed after 3 h in animals receiving the highest dose (36.5 ± 0.2 °C and 35.5 ± 0.3 °C for saline and LPS, respectively; ANOVA *P* = 0.0273; Extended Data Fig. 1e,h). The BAT temperature for the intermediate dose trended downward at 3 h post LPS injection but did not elicit a significant decrease until 7 h after injection (36.4 ± 0.1 °C and 34.5 ± 0.6 °C for saline and LPS, respectively; ANOVA *P* = 0.0455), whereas the lowest LPS dose did not cause a significant change in temperature. Tail temperature also showed a significant decrease starting at 3 h post LPS injection for both the highest and intermediate dose compared to saline (26.2 ± 0.3 °C, 24.7 ± 0.1 °C and 24.4 ± 0.2 °C for saline, high dose and intermediate dose, respectively; ANOVA *P* = 0.0013 and *P* = 0.0008 respectively; Extended Data Fig. 1f,i). The decrease in tail temperature is indicative of vasoconstriction, which suggests that blood flow to the tail does not contribute to the decrease in core temperature. Rather, reduced heat production by BAT contributes to the hypothermia after LPS treatment.

Although the qualitative behavioural response to all three doses was very similar at early time points, the higher doses of LPS resulted in effects of longer durations, with the highest dose of 2.5 mg kg^−1^ eliciting effects that were still evident 48 h post injection (Fig. 1b,d,f). By contrast, the intermediate dose (0.5 mg kg^−1^) caused significant effects for only 24 h whereas effects of the lowest dose (0.1 mg kg^−1^) began to diminish within 12 h post injection. The effect on core temperature followed a similar trend, and the time course of the hypothermic response also scaled with dose, with the highest dose inducing an average minimal core temperature of 32.3 ± 0.5 °C compared to 33.6 ± 0.7 °C for the medium dose and 35.3 ± 0.2 °C for the lowest dose (Extended Data Fig. 1g).

As the qualitative response was highly consistent for the different doses, we continued our studies of the neural mechanisms using an LPS dose of 0.5 mg kg^−1^, the intermediate dose. This dose is about 20-fold lower than the lethal dose yet, as shown above, leads to robust and coordinated behavioural and thermal responses.

## Brain-wide activity changes after LPS

**Fig. 2:**
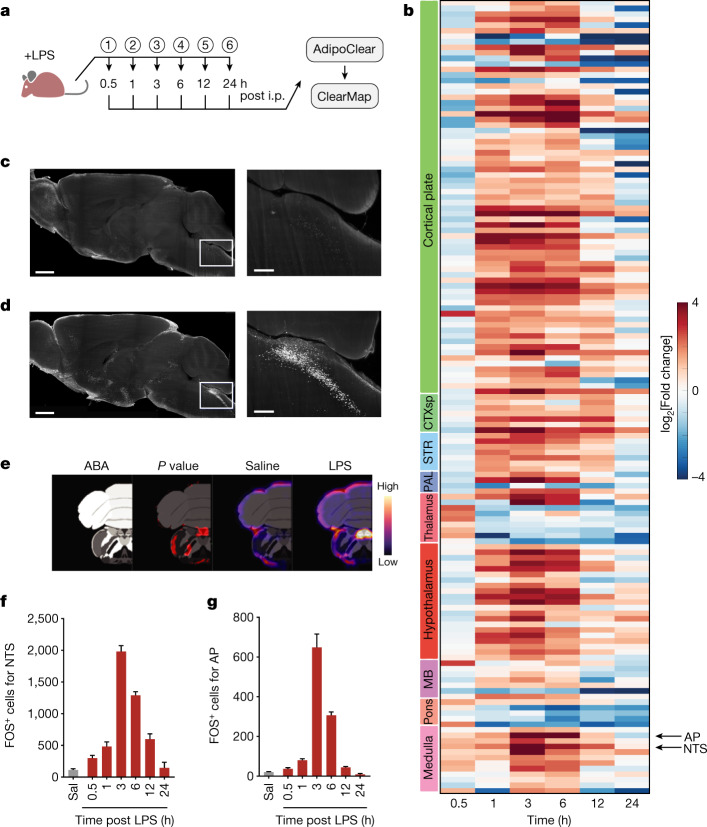
Brain-wide changes in activity during sickness behaviour. **a**, Time line for sample collection and subsequent whole-mount brain imaging of FOS. Samples (circled numbers) were collected at the indicated time points post saline or LPS (0.5 mg kg^−1^) injection. *n* = 4 for each group except for 24 h post LPS, for which *n* = 3. **b**, Heatmap of log_2_[fold change] for LPS versus saline in the indicated regions at each time point. Only regions with at least one significant change at a time point (false discovery rate-adjusted *P* < 0.05) are shown. Left colour bar, brain subdivision; CTXsp, cortical subplate; STR, striatum; PAL, pallidum; MB, midbrain; AP and NTS are indicated. **c**,**d**, Representative images of FOS expression 3 h after saline (**c**) and LPS (**d**) injection. The right panels show a higher-magnification view of the NTS–AP region outlined in the left panels. Scale bars, 1 mm (left) or 250 μm (right). **e**, From left to right: the NTS and AP in the Allen Brain Atlas (ABA), ClearMap output of significant changes (false discovery rate-adjusted *P* < 0.05, red) for post LPS (3 h) versus saline injection, ClearMap-generated average heatmap of FOS counts for saline injection (*n* = 4), and ClearMap-generated average heatmap of FOS counts for LPS injection (3 h, *n* = 4). **f**,**g**, FOS counts for the NTS (**f**) and AP (**g**), for the indicated treatments (LPS concentration, 0.5 mg kg^−1^) and time points; output from ClearMap. Error bars represent s.e.m. The mouse illustration in **a** was created using BioRender.com. Source data

**Extended Data Fig. 2:**
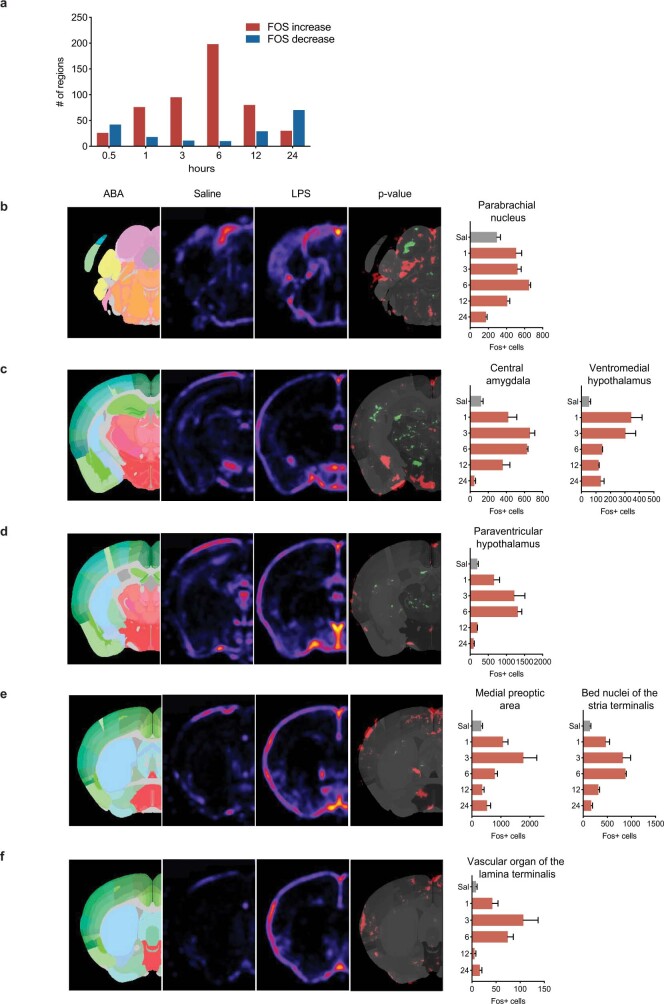
ClearMap analysis of LPS-induced FOS. **a**, Number of regions showing significant increases (red) or decreases (blue) with LPS injection at each timepoint (significance, FDR adjusted *P* < 0.05). **b**–**f**, From left to right: ClearMap outputs for Allen Brain Atlas (ABA), saline fos heatmap, LPS FOS heatmap, and p-value map (red significant increases in LPS group, green significant decreases in LPS group). Bar graphs for FOS counts are shown for each denoted region which is represented in images to the left of the graph. Source data

We next mapped the expression of the immediate early gene *Fos* in response to 0.5 mg kg^−1^ LPS after tissue clearing using whole-mount immunostaining (AdipoClear) and light-sheet imaging^17,18^. *Fos* expression was then registered to the Allen Mouse Brain Atlas (Fig. 2a). Although FOS expression can have biases for certain cell types, this approach allowed us to monitor neural activity changes brain-wide during LPS-induced sickness. We specifically quantified FOS expression at several time points post i.p. injection of LPS (0.5 h, 1 h, 3 h, 6 h, 12 h and 24 h) relative to saline control injections. Comparison of LPS-treated groups with saline-treated controls revealed significant increases of FOS in numerous brain regions (76 regions) within 1 h post injection (Extended Data Fig. 2a), including the parabrachial nucleus and bed nuclei of the stria terminalis, which have previously been reported to show increases in FOS after LPS treatment (Extended Data Fig. 2b–f). By 6 h post LPS, the number of brain regions showing increased FOS expression further increased (to nearly 200 regions) and only regions of the thalamus and pons showed significantly decreased FOS expression at this time (Fig. 2b).

**Extended Data Fig. 3:**
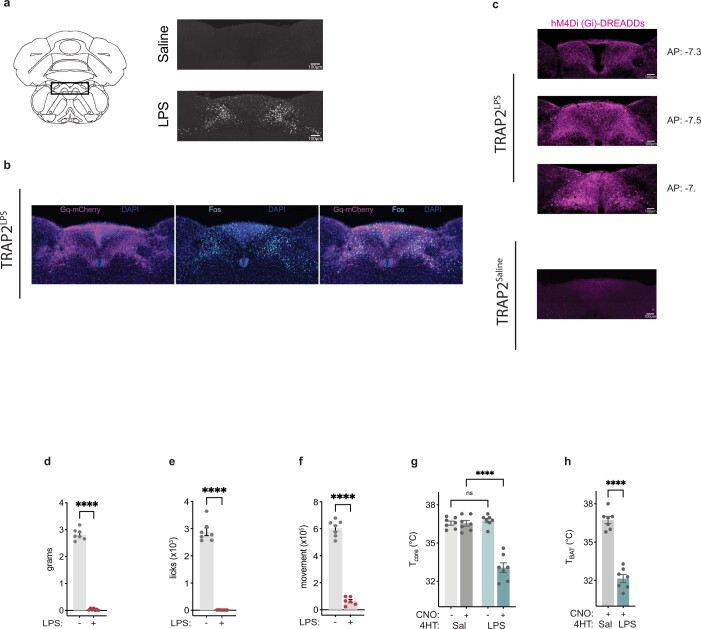
TRAP2 experiment additional information. **a**, FOS staining of NTS/AP of saline and LPS (0.5mg/kg) treated animals three hours after injection. **b**, TRAP2 labeling in NTS/AP region of LPS-labeled animals. Gq-mCherry, purple; Fos, cyan; DAPI, blue. **c**, Representative images of TRAP2 labeling in NTS/AP region of LPS labeled (top) and saline labeled (bottom) with cre-dependent Gi-DREADDs. Top three panels show 4-hydroxytamoxifen labeling with LPS for three anterior-posterior coordinates in a single animal while bottom panel shows labeling with saline. hM4Di-mCherry, magenta. **d**–**f**, food intake (**d**), water intake (**e**), and movement (**f**) for 0.5mg/kg of LPS from Fig. 1 experiment to serve as reference. **g**,**h**, Core temperature (**g**) and BAT temperature (**h**) at four hours post CNO injection for reactivation of saline labeled (dark gray) and LPS labeled (dark cyan) neurons. Saline treatment is shown for core temperature in light gray and light cyan. Two-way ANOVA core temperature *P* < 1x10^−7^; unpaired t-test BAT temperature *P* < 1x10^−7^). ****P < 0.0001 and NS, not significant (*P* > 0.05). All error bars represent s.e.m. The brain illustration in **a** was created using BioRender.com. Source data

Our objective was to identify key neurons that regulate one or more elements of the sickness response, and we reasoned that those brain regions with high levels of FOS at the earliest time points were more likely to serve this function. We thus focused on those regions that showed robust increases of FOS expression within 1–3 h post LPS injection. We found that at 3 h post LPS, the dorsal vagal complex of the brainstem consisting of the interconnected network of the AP, the NTS and the dorsal motor nucleus of the vagus showed highly significant increases in both the intensity and number of cells expressing FOS in all three components (8-fold for the NTS, 32-fold for the AP and 10-fold for the dorsal motor nucleus of the vagus; Fig. 2c–g and Extended Data Fig. 3a). Early increases in FOS expression were most prominent in the NTS with a greater than twofold increase at 1 h post LPS (Fig. 2f). As the NTS is a known target of vagal inputs and the AP is the site of a circumventricular organ with access to humoral factors, including cytokines, we next tested the role of neurons in these adjacent regions to mediate sickness behaviour by reactivating LPS-responsive neurons using TRAP2 technology^19^. The TRAP2 system enables the transient activation of the Cre recombinase in neurons that show *Fos* induction.

## The NTS–AP regulates sickness behaviours

**Fig. 3:**
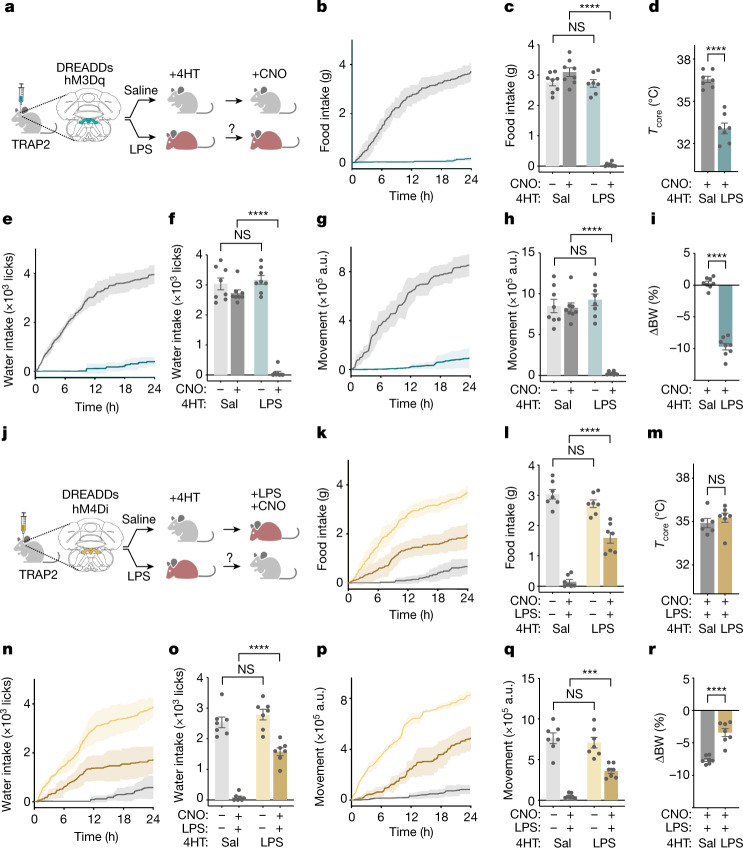
NTS–AP neurons regulate sickness behaviour. **a**, Method for TRAP2 labelling and reactivation of NTS–AP neurons using activating hM3Dq (Gq) DREADDs. Grey mouse, normal; red mouse, sick. **b**,**e**,**g**, Cumulative food intake (**b**), water intake (**e**) and movement (**g**) for 24 h after reactivation of saline-labelled (grey) and LPS-labelled (cyan) neurons. **c**,**f**,**h**, Food intake (**c**) (ANOVA *P* < 1 × 10^−7^), water intake (**f**) (ANOVA *P* < 1 × 10^−7^) and movement (**h**) (ANOVA *P* < 1 × 10^−7^) at 12 h post CNO injection for reactivation of saline-labelled (dark grey) and LPS-labelled (dark cyan) neurons; saline controls for CNO administration are also shown (light grey; light cyan). **d**,**i**, Change in core temperature (unpaired *t*-test *P* = 4.6 × 10^−6^) 4 h after reactivation (**d**) and percentage body weight (unpaired *t*-test *P* < 1 × 10^−7^) 24 h after reactivation (**i**) of saline-labelled (grey) and LPS-labelled (cyan) neurons. In all panels, saline-labelled animals, *n* = 8; LPS-labelled animals, *n* = 7.  **j**, Schematic of TRAP2 labelling and inactivation using inhibitory hM4Di (Gi) DREADDs during concurrent LPS exposure. **k**,**n**,**p**, Cumulative food intake (**k**), water intake (**n**) and movement (**p**) for 24 h after CNO-based inhibition and concurrent LPS treatment (0.5 mg kg^−1^, i.p.) of saline-labelled (grey) and LPS-labelled (dark yellow) neurons. Saline treatment of LPS-labelled neurons (light yellow) shown for reference. **l**,**o**,**q**, Food intake (**l**) (ANOVA *P* = 2.9 × 10^−7^), water intake (**o**) (ANOVA *P* = 2.1 × 10^−7^) and movement (**g**) (ANOVA *P* = 3.3 × 10^−4^) at 12 h post inhibition and concurrent LPS treatment (0.5 mg kg^−1^, i.p.) of saline-labelled (dark grey) and LPS-labelled (dark yellow) neurons; saline controls for CNO administration are also shown (light grey; light yellow). **m**,**r**, Change in core temperature (unpaired *t*-test, NS) 4 h after (**m**) and percentage body weight (unpaired *t*-test, *P* = 3.2 × 10^−5^) 24 h after (**r**) inhibition and concurrent LPS treatment of saline-labelled (grey) and LPS-labelled (yellow) neurons. In all panels, saline-labelled animals, *n* = 7; LPS-labelled animals, *n *= 7. ***P < 0.001, ****P < 0.0001 and NS, not significant (*P* > 0.05). All error bars represent s.e.m. The mouse illustrations in **a**,**j** were created using BioRender.com. Source data

We assayed the role of the NTS and AP in mediating sickness behaviours by stereotactically injecting an adeno-associated virus (AAV), with double-floxed inverted orientation (DIO), containing a Cre-dependent excitatory (Gq) DREADD fused to mCherry (AAV-DIO-Gq–mCherry), into the NTS–AP region of TRAP2 animals (Fig. 3a). TRAP2 mice express a tamoxifen-inducible Cre recombinase knocked into the *Fos* locus (FOS^2A-iCreERT2^)^19,20^. Three weeks after the AAV injections, 4-hydroxytamoxifen (4HT) and LPS (0.5 mg kg^−1^) were simultaneously injected, leading to the transient expression of Cre (about 6 h)^19^ in neurons with FOS induction, thus ‘TRAPing’ activating DREADDs in those cells that had been activated by LPS (Fig. 3a and Extended Data Fig. 3a,b). After a recovery period of a further 3 weeks, clozapine *N*-oxide (CNO) or saline was injected intraperitoneally, and food and water intake, locomotor activity, temperature and body weight were measured. Note that with this method the NTS–AP neurons that had been activated by LPS are reactivated weeks after the effects of the LPS have dissipated (typically 24–48 h for the 0.5 mg kg^−1^ dose, see above) by which time the behaviour of the animal has returned to normal.

As for LPS treatment, reactivation of the LPS-TRAPed neurons in the NTS–AP region markedly reduced food intake, water intake and movement within 30 min of CNO injection. By 12 h, CNO-treated animals consumed negligible amounts of food (0.05 ± 0.02 g and 3.10 ± 0.14 g, respectively; ANOVA *P* < 1 × 10^−7^) and water (69.75 ± 52.56 licks and 3,170.38 ± 146.81 licks, respectively; ANOVA *P* < 1 × 10^−7^) relative to controls (Fig. 3b,c,e,f and Extended Data Fig. 3d,e). They also showed a marked diminution of locomotor activity (25,543.58 ± 6,925.92 a.u. and 926,934.37 ± 71,293.37 a.u., respectively; ANOVA *P* < 1 × 10^−7^; Fig. 3g,h and Extended Data Fig. 3f). As for LPS, there was also a reduction in core (36.6 ± 0.2 °C and 33.1 ± 0.4 °C for saline- and LPS-labelled neurons, respectively; unpaired *t*-test *P* = 4.6 × 10^−6^) and BAT (36.7 ± 0.3 °C and 32.1 ± 0.3 °C, unpaired *t*-test *P* < 1 × 10^−7^; unpaired *t*-test *P* < 1 × 10^−7^; Fig. 3d and Extended Data Fig. 3g,h) temperature 4 h after CNO injection and body weight 24 h after CNO injection (−9.7 ± 0.5%). Saline injection of LPS-TRAPed animals had no discernible effect on these parameters nor did reactivation of neurons TRAPed after saline injections (Fig. 3b–i). These findings show that neurons in the NTS–AP region are sufficient for eliciting each of the different components of sickness behaviour that we followed.

We next tested whether NTS–AP neurons were necessary for the induction of sickness behaviours by inhibiting these populations during LPS treatment. In this experiment, the same protocol using TRAP2 mice was used except that in this case an inhibitory (Gi) DREADD fused to mCherry (AAV-DIO-Gi–mCherry) was injected into the NTS–AP (Fig. 3j and Extended Data Fig. 3c). The necessity of NTS–AP neurons for sickness behaviour was evaluated by subsequently inhibiting the LPS-activated neurons concomitant with LPS treatment. Inhibition of LPS-TRAPed neurons significantly diminished the response to an LPS injection (0.5 mg kg^−1^) with a significantly reduced effect of LPS on feeding (1.60 ± 0.18 g and 0.15 ± 0.07 g, respectively; ANOVA *P* = 2.9 × 10^−7^), drinking (1,576.29 ± 130.07 licks and 82.71 ± 44.37 licks, respectively; ANOVA *P* = 2.1 × 10^−7^), movement (360,694.86 ± 31,672.62 a.u. and 50,245.00 ± 11,865.28 a.u., respectively; ANOVA *P* = 3.3 × 10^−4^) and body weight (−3.4 ± 0.6% and −7.6 ± 0.2%, respectively; unpaired *t*-test *P* = 3.2 × 10^−5^; Fig. 3k–r). By contrast, inhibition of these NTS–AP neurons did not alter the temperature response to LPS treatment (Fig. 3m) 4 h post treatment. These data show that activation of neurons in the NTS–AP region is necessary for eliciting the behavioural responses to LPS, whereas the thermal responses seem to be mediated by other sites.

## Sickness-activated NTS–AP neuronal types

**Fig. 4:**
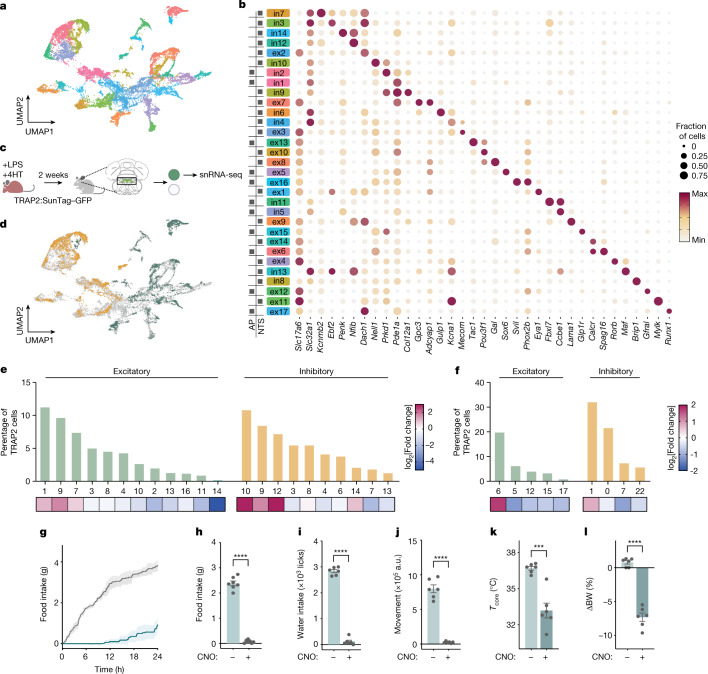
Identification of LPS-activated cell types in the NTS–AP. **a**, A uniform manifold approximation and projection (UMAP) plot of 7,824 and 4,443 neuronal nuclei from the NTS and the AP, respectively, from 20 mice. **b**, Expression of the indicated marker genes across identified clusters, with the colours of the clusters the same as in the UMAP in **a**. The size of the circle represents the fraction of cells in each cluster expressing the marker gene; the colour of the circle represents the minimum to maximum level of gene expression for each gene. The boxes on the left indicate the presence of the cluster in the AP or NTS. **c**, Method for TRAP2 labelling with LPS (0.5 mg kg^−1^, i.p.) in INTACT mice for subsequent processing for snRNA-seq. **d**, UMAP of TRAPed nuclei of excitatory (TRAP2^VGLUT2^, orange) or inhibitory (TRAP2^VGAT^, green) neurons superimposed on a UMAP of all neuronal nuclei from NTS–AP (grey). **e**,**f**, Percentage of TRAP2 neurons within each indicated cluster (excitatory, green; inhibitory, orange) from NTS (**e**) and AP (**f**). The heatmaps below the plots indicate log_2_[fold change] TRAPed nuclei for each cluster relative to the baseline sequencing in **a**. **g**–**l**, Activation of VGLUT2 neurons in the NTS–AP using activating (hM3Dq) DREADDs in VGLUT2–Cre mice. **g**, Cumulative food intake for 24 h post activation (saline, grey; CNO, cyan). **h**–**j**, Food intake (**h**, paired *t*-test *P* = 8.3 × 10^−7^), water intake (**I**, paired *t*-test *P* < 1 × 10^−7^) and movement (**j**, paired *t*-test *P* = 4.1 × 10^−5^) at 12 h post activation of saline-treated (light cyan) or CNO-treated (dark cyan) animals. **k**,**l**, Change in core temperature (paired *t*-test *P* = 8.6 × 10^−4^) 4 h after (**k**) and percentage body weight (paired *t*-test *P* = 8.0 × 10^−5^) 24 h after (**l**) activation of saline-treated (light cyan) or CNO-treated (dark cyan) animals. **g**–**l**, Saline-treated animals, *N* = 7; CNO-treated animals, *n* = 7. ***P < 0.001, ****P < 0.0001. All error bars represent the s.e.m. The mouse illustration in **c** was created using BioRender.com. Source data

**Extended Data Fig. 4:**
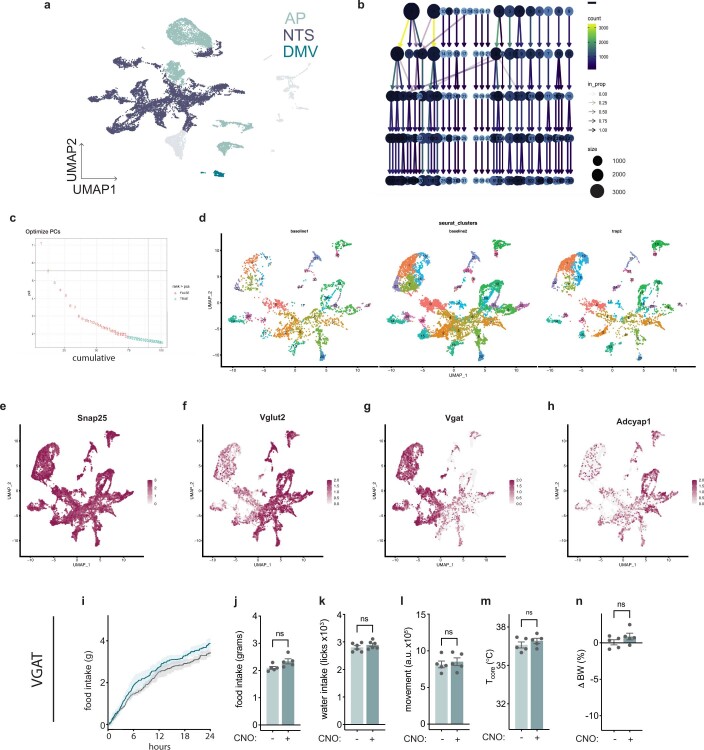
snRNA-seq metrics and excitatory vs. inhibitory functional testing. **a**, UMAP plot of all nuclei sequenced after removal of  < 2000 gene nuclei. AP, NTS, and DMV are denoted in separate colors, light grey is used for neurons outside of these regions. **b**, Output from clustree for clustering used. **c**, Elbow plot used for determining principal components to use for clustering. **d**, UMAP plots of the two baseline sequencing datasets and the TRAP2-seq dataset. **e**–**h**, UMAP plots colored by normalized expression of the denoted gene generated by R. **i**–**n**, Activation of Vgat neurons in the NTS/AP using activating (hM3Dq) DREADDs in Vgat-cre mice. **i**, Cumulative food intake for 24 h post activation; saline, gray; CNO, cyan. **j**–**l** Food intake (**j**, paired t-test NS), water intake (**k**, paired t-test NS), and movement (**l**, paired t-test NS) at 12 h post activation of saline treated (light cyan) or CNO treated (dark cyan) animals. **m**-**n**, Change in core temperature (**m**, paired t-test NS) four hours after and percent body weight (**n**, paired t-test NS) 24 h after activation of saline treated (light cyan) or CNO treated (dark cyan) animals. **i**–**n**, Saline treated animals, N = 5; CNO treated, N = 5. NS, not significant (P > 0.05). All error bars represent s.e.m Source data

The NTS and AP are comprised of a heterogeneous group of neurons that can serve different functions, with the NTS primarily relaying afferent vagal signals^21^ and the AP, the site of a circumventricular organ, sensing humoral signals^22,23^. We next set out to map the effects of LPS onto individual cell type(s) in these regions with the aim of defining which cell types are responsible for the response. We first performed single-nucleus RNA sequencing (snRNA-seq) from the entire NTS–AP by isolating individual nuclei immunolabelled with NeuN, a neuronal nuclear marker, followed by fluorescence-activated cell sorting. In addition, we used hashing^24^ to differentiate between neurons in the NTS versus AP. Using this approach, high-quality sequencing data (>2,000 unique genes detected per cell) were obtained from 7,824 neurons in the NTS and 4,443 neurons in the AP (Fig. 4a and Extended Data Fig. 4a). Louvain clustering identified 12 excitatory and 10 inhibitory clusters in the NTS region (Fig. 4b and Extended Data Fig. 4b–g). In the AP, there were five excitatory and four inhibitory neuronal clusters (Fig. 4b and Extended Data Fig. 4b–g). On the basis of co-expression of marker genes in the different clusters, the atlas of cell types we identified in the NTS and AP regions was well aligned with similar data in recent reports^25,26^.

We next set out to identify the subpopulations in the NTS–AP that are activated by LPS by performing snRNA-seq after treating TRAP2 mice mated to the INTACT mouse line with LPS (Fig. 4c). INTACT mice carry a Cre-dependent knock-in of a Sun1–GFP fusion protein that is expressed exclusively on the nuclear membrane. Sun1–GFP-expressing nuclei can then be sorted by fluorescence-activated cell sorting and sequenced. Animals carrying both transgenes were treated with 4HT and LPS to activate Cre, leading to expression of Sun1–GFP in those neurons that were previously activated by LPS. Two weeks later, by which time the animals’ behaviour had returned to normal, GFP^+^ nuclei were isolated by fluorescence-activated cell sorting and processed in the same manner as described above. This yielded 2,923 neuronal nuclei from the NTS and 1,841 neuronal nuclei from the AP. This dataset, henceforth referred to as the TRAP2-seq dataset, was aligned to the aforementioned atlas of NTS–AP cell types (Fig. 4d). By comparing the TRAP2-seq data to the baseline-seq data, we identified those cell types that had previously been activated by LPS. For each, we calculated their enrichment as the percentage of the total number of cells in the TRAP2-seq versus the percentage in the baseline snRNA-seq datasets. The data for each of the cell types that were enriched are represented as a series of clusters and a defining marker is denoted for each (Fig. 4e,f). We found significant enrichment of four excitatory clusters all expressing vesicular glutamate transporter 2 (VGLUT2): ex1 expressing EYA1, ex9 expressing LAMA1, ex7 expressing GPC3 and ADCYAP1, and ex6 expressing SPAG16 (enrichment of 0.73, 1.38, 0.39 and 1.51 log_2_[fold change], respectively). We also identified four inhibitory clusters that express vesicular GABA transporter (VGAT): in10 expressing NELL1, in9 expressing COL12A1, in12 expressing NFIB and in2 expressing PRKD1 (2.54, 1.35, 2.58 and 0.60 log_2_[fold change], respectively; Fig. 4e,f). We next tested which of these neural populations are responsible for one or more components of sickness behaviour.

## ADCYAP1 neurons cause sickness behaviours

We began by evaluating whether the excitatory or inhibitory neuron clusters mediate sickness behaviour using VGLUT2–Cre, the only glutamate transporter expressed in all excitatory clusters, and VGAT–Cre mice. An AAV containing a Cre-dependent excitatory (Gq) DREADD fused to mCherry (AAV-DIO-Gq–mCherry) was stereotactically injected into the NTS–AP of either of these Cre-expressing mouse lines. Chemogenetic activation of the excitatory NTS–AP^VGLUT2+^ cells elicited a strong reduction of food intake (2.39 ± 0.09 g and 0.07 ± 0.03 g for saline- and CNO-treated animals, respectively; paired *t*-test *P* = 8.3 × 10^−7^), water intake (2,835.50 ± 61.49 licks and 113.67 ± 57.29 licks for saline and CNO respectively; paired *t*-test *P* < 1 × 10^−7^) and locomotor activity (798,016.00 ± 56,553.22 a.u. and 26,287.00 ± 5,397.98 a.u. for saline- and CNO-treated animals, respectively; paired *t*-test *P* = 4.1 × 10^−5^) for 24 h (Fig. 4g–j) and also reduced core temperature (36.7 ± 0.2 °C and 33.2 ± 0.6 °C at 4 h; paired *t*-test *P* = 4.9 × 10^−3^) and body weight (0.9 ± 0.4% and −7.3 ± 0.6% for saline- and CNO-treated animals, respectively; paired *t*-test *P* = 8.0 × 10^−5^; Fig. 4k,l). As was seen for 0.5 mg kg^−1^ LPS, a response was first observed within 1 h and persisted for about 24 h. By contrast, activation of the inhibitory NTS–AP^VGAT+^ neurons failed to show a significant effect on these responses (Extended Data Fig. 4i–n).

**Fig. 5:**
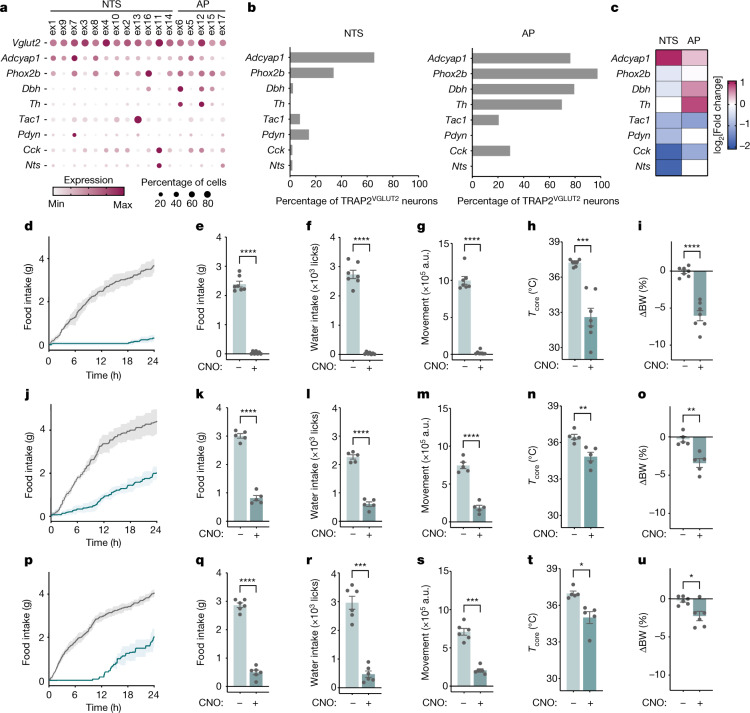
NTS–AP ADCYAP1 neurons drive sickness behaviours. **a**, Expression of the indicated marker genes across TRAP2-enriched clusters. The size of the circle represents the fraction of cells in each cluster expressing the marker gene; the colour of the circle represents the minimum to maximum level of gene expression for each gene. **b**, Percentage of TRAP^VGLUT2^ neurons labelled by each marker gene in the NTS (left) and the AP (right). **c**, log_2_[fold change] TRAPed nuclei for each marker gene relative to baseline sequencing in the NTS or AP. **d**–**u**, Activation of ADCYAP1 (**d**–**I**, *n* = 7), DBH (**j**–**o**, *n* = 5) or PHOX2B (**p**–**u**, *n* = 6) neurons in the NTS–AP using activating hM3Dq (Gq) DREADDs in corresponding Cre lines. **d**,**j**,**p** Cumulative food intake for 24 h post activation; saline, grey; CNO, cyan. **e**–**g**,**k**–**m**,**q**–**s**, Food intake (**e**, paired *t*-test *P* = 2.8 × 10^−7^; **k**, paired *t*-test *P* = 2.6 × 10^−5^; **q**, paired *t*-test *P* = 2.1 × 10^−6^), water intake (**f**, paired *t*-test *P* = 1.8 × 10^−6^; **l**, paired *t*-test *P* = 2.2 × 10^−6^; **r**, paired *t*-test *P* = 1.5 × 10^−4^) and movement (**g**, paired *t*-test *P* = 3.6 × 10^−6^; **m**, paired *t*-test *P* = 4.0 × 10^−6^; **s**, paired *t*-test *P* = 1.4 × 10^−4^) at 12 h post CNO injection for activation of saline-treated (grey) or CNO-treated (cyan) animals. **h**,**i**,**n**,**o**,**t**,**u**, Change in core temperature 4 h after (**h**, paired *t*-test *P* = 5.0 × 10^−4^; **n**, paired *t*-test *P* = 3.0 × 10^−3^; **t**, paired *t*-test *P* = 4.1 × 10^−2^) and percentage body weight 24 h after (**i**, paired *t*-test *P* = 6.8 × 10^−5^; **o**, paired *t*-test *P* = 2.5 × 10^−3^; **u**, paired *t*-test *P* = 2.0 × 10^−2^) activation of saline-treated (grey) or CNO-treated (cyan) animals. *P < 0.05, **P < 0.01, ***P < 0.001, ****P < 0.0001 and NS, not significant (*P* > 0.05). All error bars represent s.e.m. Source data

**Extended Data Fig. 5:**
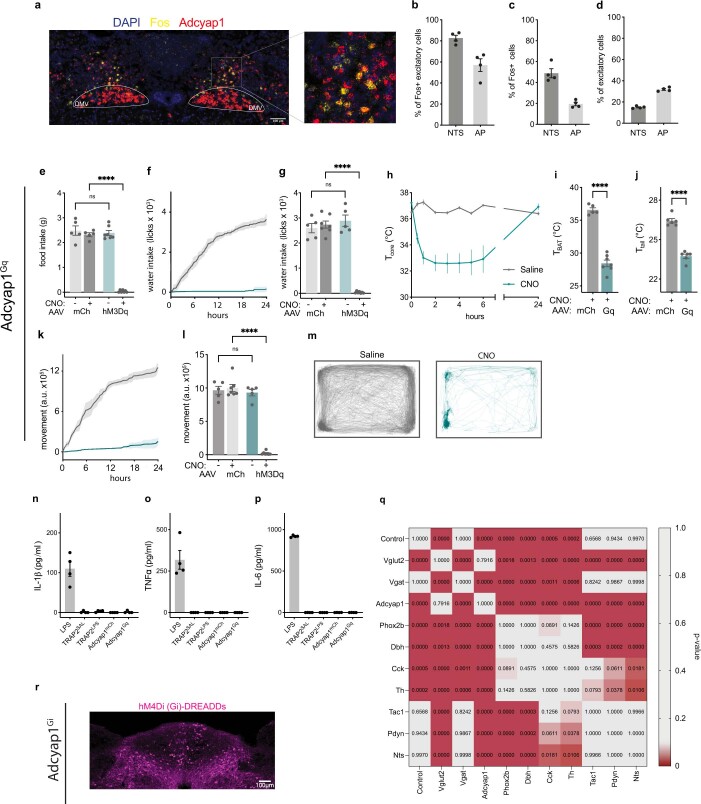
Functional characterization of cell types in NTS/AP region. **a**, Representative RNAscope image of DAPI (blue), Fos (yellow), and Adcyap1 (red) staining in the NTS/AP of animals two hours after treatment with 0.5mg/kg of LPS. **b**, Quantification of % of Fos+ excitatory cells coexpressing Adcyap1 in the NTS and AP after LPS treatment. **c**, Quantification of % of Fos+ cells coexpressing Adcyap1 in the NTS and AP after LPS treatment. **d**, Quantification of % of all excitatory cells expressing Adcyap1 in the NTS and AP. **e**,**g**,**i**, Total 12 h food intake (**e**), water intake (**g**), and movement (**i**) for mCherry virus injected animals (grey) and activating hM3Dq DREADDs injected animals (cyan). **f**,**k**, Cumulative water intake (**f**) and movement (**k**) for saline (grey) or CNO (cyan) injection. **m**, Movement trace for first 24 h post saline (grey) or CNO (cyan) for representative mouse. **h**, Core temperature time course for 24 h after injection of saline (grey) or CNO (cyan). **i**,**j**, BAT temperature (i) and tail temperature (j) four hours after injection of CNO for mCherry virus injected animals (grey) and activating hM3Dq DREADDs injected animals (cyan). Unpaired t-test BAT temperature *P* < 1x10^−7^; unpaired t-test tail temperature P = 3.8x10^−7^. **n**-**p**, Serum levels of IL-1β (**n**), TNFα (**o**), and IL-6 (**p**), after LPS treatment, and CNO treatment for: saline labeled TRAP2 cells expressing cre dependent Gq DREADDs; LPS labeled TRAP2 cells expressing cre dependent Gq DREADDs, Adcyap1-cre injected with cre dependent mCherry, and Adcyap1-cre injected with cre dependent Gq-DREADDs. **q**, P-value statistics from two-way ANOVA comparing food intake of cre lines to one another from Fig. 5v. **r**, Representative image of an Adcyap1-cre injected with cre-dependent Gi-DREADDs. hM4Di-mCherry, magenta. ****P < 0.0001 and NS, not significant (*P* > 0.05). All error bars represent s.e.m. Source data

We next assessed the role of the different excitatory neural clusters using mouse lines expressing Cre under the control of markers expressed in that cluster(s). We began by using ADCYAP1^+^–Cre mice because ADCYAP1^+^ clusters account for 65.5% of activated NTS neurons (ex1, ex9, ex7 and ex3) and 76.4% of activated AP neurons in the TRAP2-seq dataset of LPS-activated cells (enrichment of 1.01 log_2_[fold change] and 0.33 log_2_[fold change], respectively; Fig. 5b,c). We confirmed the distribution of ADCYAP1 using in situ hybridization, which showed that ADCYAP1^+^ cells account for 82.7 ± 2.7% and 57.0 ± 6% of FOS^+^ excitatory (VGLUT2^+^) cells after LPS treatment in the NTS and AP, respectively, whereas at the baseline, ADCYAP1^+^ neurons account for only 15.0 ± 0.5% and 31.9 ± 0.9% of excitatory neurons in the NTS and AP, respectively (Extended Data Figs. 4h and 5a–d). To target NTS–AP ^ADCYAP1+^ neurons, an AAV containing a Cre-dependent excitatory (Gq) DREADD fused to mCherry (AAV-DIO-Gq–mCherry) was injected into the NTS–AP of ADCYAP1–Cre mice. Chemogenetic activation of NTS–AP^ ADCYAP1+^ neurons with CNO elicited strong reductions in feeding (−91.6 ± 3.2%, paired *t*-test *P* = 2.8 × 10^−7^), drinking (−92.2 ± 3.8%; paired *t*-test *P* = 1.8 × 10^−6^) and movement for 24 h (−90.0 ± 2.1%; paired *t*-test *P* = 3.6 × 10^−6^) relative to saline-injected controls (Fig. 5d–g and Extended Data Fig. 5e,f,k–m). CNO also led to a pronounced reduction in core temperature (36.6 ± 0.3 °C and 28.4 ± 0.5 °C, respectively; paired *t*-test *P* = 2 × 10^−7^), BAT, and tail temperature and body weight (−6.0 ± 0.7%; paired *t*-test *P* = 6.8 × 10^−5^) when measured 4 h post treatment (Fig. 5i and Extended Data Fig. 5h–j).

**Extended Data Fig. 6:**
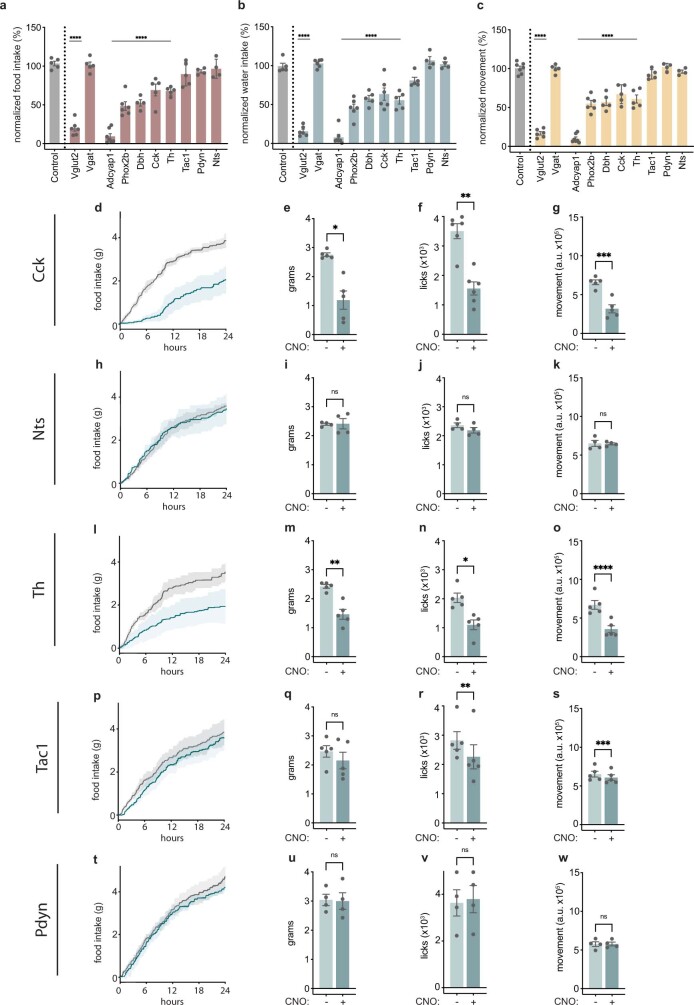
Characterization of additional cre lines available for TRAP2-seq cell types. **a**–**c**, Food intake (**a**), water intake (**b**), and movement (**c**) for at 24 h post CNO based activation of listed cre lines normalized to saline treatment for respective cre line. **d**,**h**,**l**,**p**,**t**, Cumulative 24 h food intake for denoted cre lines after chemogenetic activation. **e**,**I**,**m**,**q**,**u**, Food intake for denoted cre lines for 12 h after chemogenetic activation. **f**,**j**,**n**,**r**,**v**, Water intake for denoted cre lines for 12 h after chemogenetic activation. **g**,**k**,**o**,**s**,**w**, Movement for denoted cre lines for 12 h after chemogenetic activation. *P < 0.05, **P < 0.01, ***P < 0.001, ****P < 0.0001 and NS, not significant (*P* > 0.05). All error bars represent s.e.m. Source data

ADCYAP1 marks neurons in both the NTS and AP regions. To define the relative contributions of AP versus NTS, we then tested the effect of activating just the AP using dopamine-β-hydroxylase (DBH)–Cre, tyrosine hydroxylase (TH)–Cre and cholecystokinin (CCK)–Cre lines, each of which labels LPS-activated neurons predominantly in this region relative to the NTS. DBH and TH (ex6 and ex12) are expressed in an overlapping set of clusters in the AP (ex6, ex12, ex15 and ex16; Fig. 5a) whereas CCK is expressed in a separate set of AP clusters (ex11, ex5 and ex12; Fig. 5a). DBH–Cre, TH–Cre and CCK–Cre mice label an aggregate of 97.5% of the activated cells in the AP with only minimal labelling of LPS-activated neurons in the NTS (2.3%, 0% and 1.8%, respectively). Although activation of each of these three neural populations in Cre-expressing mice reduced food intake, water intake and movement, the effect was significantly lower than observed after activation of ADCYAP1^+^ neurons (DBH, −47.5% ± 4.2%; TH, −32.2% ± 2.4%; and CCK, −30.9% ± 7.6% for 24 h food intake, compared to 91.6 ± 3.2% for each using ADCYAP1–Cre mice; Fig. 5j–o and Extended Data Fig. 6). In addition, the effects of activating DBH, TH and CCK neurons were limited to the first 6–12 h in contrast to the ≈24 h duration seen after activation of ADCYAP1^+^ neurons.

These data suggested that ADCYAP1 neurons in the NTS play a greater role to mediate sickness behaviour compared to neurons in the AP. We studied this further using PHOX2B–Cre mice. PHOX2B is expressed in the same LPS-activated cell types in AP as ADCYAP1 (97.5% and is thus expressed in the same clusters that express DBH, TH and CCK), but is expressed only in a subset of the NTS clusters. In the NTS, PHOX2B is expressed in 34.0% of the LPS-activated NTS neurons corresponding to only some of the ADCYAP1 clusters (ex1, ex7 and ex8) but not others (ex9). Thus, as PHOX2B is expressed in nearly all activated AP neurons, differences in the effects of activation of ADCYAP1 versus PHOX2B neurons would suggest that the effect is mediated by neurons in the NTS in those clusters that express ADCYAP1 but not PHOX2B (that is, NTS ADCYAP1^+^PHOX2B^−^ neurons in clusters 1, 9, 7 and 8, see below).

An AAV containing a Cre-dependent excitatory (Gq) DREADD fused to mCherry (AAV-DIO-Gq–mCherry) was injected into the NTS–AP of PHOX2B–Cre mice. As for activation of CCK, TH and DBH neurons, CNO treatment of the PHOX2B–Cre mice led to a smaller effect than did ADCYAP1^+^ neural activation. Thus, although there were significant reductions in food intake, water intake and movement (−51.5 ± 5.5%, −54.5 ± 5.0% and −44.9 ± 4.1% for 24 h, respectively; Fig. 5p–s), the effect was significantly smaller than was observed after ADCYAP1 activation and of shorter duration (*P* = 2.9 × 10^−6^ comparing food intake of ADCYAP1–Cre and PHOX2B–Cre mice; Extended Data Fig. 5q). The effects on temperature were also lower in magnitude than seen after activation of ADCYAP1^+^ neurons, with a significantly shorter reduction in core temperature (37.0 ± 0.2 °C and 35.0 ± 0.5 °C for saline- and CNO-treated animals, respectively, *P* = 0.0414; Fig. 5t) and body weight (−2.3 ± 0.6%, *P* = 2.3 × 10^−2^; Fig. 5u). Activation of ADCYAP1 neurons also failed to alter the levels of interleukin-1β (IL-1β), tumour necrosis factor (TNF) and IL-6 (Extended Data Fig. 5n–p). Together these data thus suggest the possibility that a significant portion of sickness behaviour is mediated by ADCYAP1^+^ neurons in NTS clusters 1, 9, 7 and 8, which do not express PHOX2B. To further refine this, we activated further NTS neural populations, including those expressing prodynorphin (expressed in cluster ex7), tachykinin (expressed in cluster ex13) and neurotensin (expressed in cluster ex11). Activation of these neurons using (Gq)-DREADD injection into specific Cre-expressing lines followed by CNO treatment failed to show alterations of food and water intake, movement or temperature. Taken together, these data suggest that a subset of ADCYAP1^+^ neurons in NTS in clusters ex1, ex8 or ex9, which do not express PHOX2B, are required for induction of the full sickness behaviour response. Next, we administered LPS to ADCYAP1–Cre, PHOX2B–Cre and DBH–Cre mice while inhibiting these populations using chemogenetics. As mentioned above, DBH is expressed only in AP and not NTS, thus providing a means for distinguishing between the contributions of these two adjacent regions. These loss-of-function studies are of critical importance for establishing the normal role of these neurons to mediate the response to LPS.

**Fig. 6:**
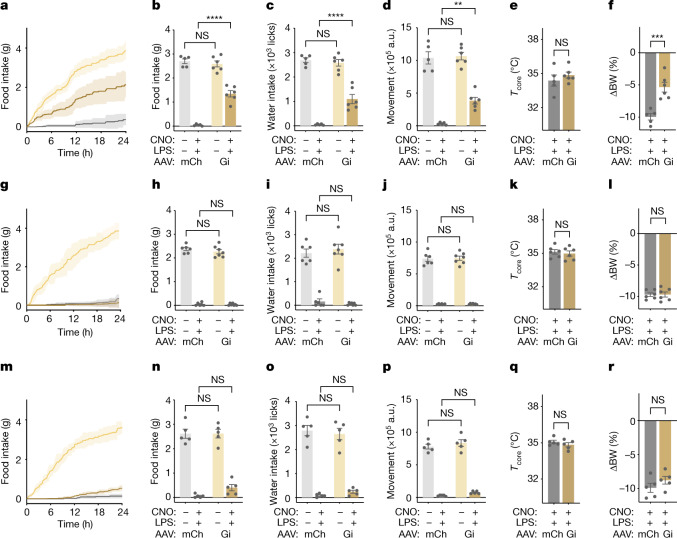
NTS–AP ADCYAP1 neurons are necessary for sickness behaviours. **a**,**g**,**m**, Inhibition of ADCYAP1 (**a**, *n *= 6), DBH (**g**, *n* = 7) or PHOX2B (**m**, *n* = 6) NTS–AP neurons following LPS injection. Cumulative food intake after treatment with saline (light yellow) or CNO and LPS (dark yellow) in animals expressing inhibitory DREADDs is shown. A further control group of animals transduced with a control AAV expressing mCherry (ADCYAP1–Cre *n* = 5; DBH–Cre, *n *= 6; PHOX2B–Cre, *n* = 5) and treated with CNO and LPS is also shown for comparison (grey). **b**–**f**, ADCYAP1 inhibition; **h**–**l**, DBH inhibition; **n**–**r**, PHOX2B inhibition: food intake (**b**, two-way ANOVA *P* = 2.5 × 10^−7^; **h**, two-way ANOVA NS; **n**, two-way ANOVA NS), water intake (**c**, ANOVA *P* = 6.6 × 10^−5^; **i**, ANOVA NS; **o**, ANOVA NS) and movement (**d**, ANOVA *P* = 1.4 × 10^−3^; **j**, ANOVA NS; **p**, ANOVA NS) at 12 h post CNO injection for inactivation. Animals transduced with control AAV (mCherry (mCh)) and treated with saline (light grey) or concurrent LPS and CNO (dark grey) as well as animals transduced with inhibitory DREADDs (Gi) and treated with saline (light yellow) or concurrent LPS and CNO (dark yellow) are shown. **e**,**f**, ADCYAP1 inhibition; **k**,**l**, DBH inhibition; **q**,**r**, PHOX2B inhibition: change in core temperature 4 h after (**e**, unpaired *t*-test NS; **k**, unpaired *t*-test NS; **q**, unpaired *t*-test NS) and percentage body weight 24 h after (**f**, unpaired *t*-test *P* = 9.0 × 10^−4^; **l**, unpaired *t*-test NS; **r**, unpaired *t*-test NS) inactivation (CNO) and concurrent LPS treatment in control AAV animals (mCh, dark grey) and animals expressing an inhibitory DREADD (Gi, dark yellow). **P < 0.01, ***P < 0.001, ****P < 0.0001 and NS, not significant (*P* > 0.05). Source data

An inhibitory (Gi) DREADD was injected into the NTS–AP of ADCYAP1–Cre mice, PHOX2B–Cre mice or DBH–Cre mice followed by simultaneous treatment with LPS ± CNO. Similar to the results seen using TRAP2 mice, the effects of LPS on food intake were significantly decreased by inhibition of NTS–AP^ ADCYAP1^ neurons (1.35 ± 0.13g and 0.04 ± 0.02 g for Gi and mCherry mice, respectively; ANOVA *P* = 2.5 × 10^−7^), water intake (1,100.50 ± 187.99 licks and 52.40 ± 13.00 licks for Gi and mCherry mice, respectively; ANOVA *P* = 6.6 × 10^−5^) and locomotor activity (383,720.50 ± 51,177.01 a.u. and 33,395.20 ± 8,191.87 a.u. for Gi and mCherry mice, respectively; ANOVA *P* = 1.4 × 10^−3^), with effects being strongest in the first 12 h after injection (Fig. 6). Furthermore, similarly to TRAP2-based inhibition of sickness-activated neurons in this region, NTS–AP ^ADCYAP1^ inhibition dimished the body weight reduction induction by LPS (−5.3 ± 0.8% and −10.0 ± 0.5% for Gi and mCherry mice, respectively; unpaired *t*-test *P* = 9.0 × 10^−4^). Although activation of NTS–AP ^ADCYAP1^ was sufficient to reduce the core and BAT temperature (Fig. 5h and Extended Data Fig. 5h,i), inhibition of these neurons during LPS-induced sickness behaviour did not abrogate the reduction in core temperature (Fig. 6e). Inhibition of NTS–AP^ ADCYAP1^ neurons without concomitant LPS treatment failed to reveal an effect on the behaviours under study. The behavioural response to both activation of NTS–AP^ ADCYAP1^ neurons and inactivation of these neurons during LPS treatment was nearly identical to the findings of analogous experiments of LPS-TRAPed neurons (Fig. 3).

By contrast, the effect of LPS treatment was unaltered by DREADD inhibition of DBH or PHOX2B neurons. Thus, after injection of an inhibitory (Gi) DREADD into the NTS–AP of DBH–Cre or PHOX2B–Cre mice, CNO failed to mitigate the reduction in food and water intake, locomotor activity and temperature seen after LPS injection (Fig. 6g–l). Thus, although activation of DBH^+^ and PHOX2B^+^ neurons is able to induce sickness behaviours with smaller effects, these neurons, which label nearly all of the neurons in the AP, are not required for LPS-induced sickness behaviour. By contrast, neurons positive for ADCYAP1, which is expressed in the NTS and AP, are both sufficient and necessary for the induction of the full behavioural response to LPS and thus play a key role in generating the sickness response.

## Discussion

Bacterial, viral and fungal infections induce a pleiotropic set of physiologic and behavioural responses that are essential for an animal’s recovery^1,27^. Thus ‘sickness’ is not just a consequence of an infection but rather a component of an adaptive response that includes alterations of several motivated behaviours, including eating^4,28^, drinking and locomotion^11^, as well as a reduction of core temperature^8,12,29^. Despite their prominent behavioural components, the neural mechanisms that mediate these responses have not been determined. Moreover, as there are several distinct elements, it was not clear whether the various responses are controlled by a distributed, parallel network with different cells regulating different aspects or whether a single population could coordinate several different elements of the response.

In this study we administered LPS, a well-established means for inducing sickness behaviour^15^, and used an unbiased approach to map the neuronal response. We found that neurons in the NTS–AP of the brainstem are rapidly activated by LPS and catalogued these active populations using snRNA-seq. Further studies revealed that brainstem neurons expressing ADCYAP1 are both sufficient and necessary for the full behavioural response to LPS injection. Thus, activation of these neurons reduces food and water intake, locomotor activity and temperature, and inhibiting them significantly weakens all responses except for the effect on temperature. By contrast, we find that although activating other brainstem populations, including neurons expressing PHOX2B, which labels some NTS cells and nearly all AP cells, and DBH, which exclusively labels LPS-activated AP neurons, can reduce food and water intake and locomotor activity, the magnitude and duration of the response is not as robust. Moreover, inhibition of ADCYAP1^+^ neurons significantly decreases the response to LPS whereas inhibiting PHOX2B and DBH neurons had no effect. Although similar to ADCYAP1^+^ neurons, these other neuronal populations can elicit behavioural responses, but less robustly, and thus they are not necessary for the response to LPS. These data identify ADCYAP1^+^ neurons in the brainstem as a critical component of several aspects of the sickness response and further suggest that a subset of ADCYAP1^+^ neurons in the NTS mediate this response. However, confirmation of this will require the generation of new Cre lines for markers that are expressed exclusively in the NTS, including EYA1 (ex1), LAMA1 (ex9) and GAL (ex8). Thus, although the data raise the possibility that neurons in the NTS rather than the AP mediate the sickness response, further studies will be necessary to confirm this.

We also find that inhibiting ADCYAP1 neurons in the brainstem diminishes but does not completely ablate the behavioural response to LPS and has no effect on the thermal response. These findings further suggest that other populations in the central nervous system also mediate quantitative aspects of sickness behaviours and are essential for the temperature response. Numerous other brain regions were acutely activated in response to LPS, including the preoptic area, which regulates body temperature, and further studies will be necessary to establish their contribution to the sickness response, and in particular their effect on temperature. For example, neurons in other circumventricular organs besides the AP, including the organum vasculosum of the lamina terminalis, the subfornical organ and the median eminence, may also sense circulating signals induced during immune responses. These regions also show increases in FOS after LPS treatment (Extended Data Fig. 2), suggesting possible involvement in regulation of sickness behaviours. Indeed, previous studies have shown that the subfornical region contributes to the induction of fever after infection^30^, which is in agreement with the inability of NTS–AP inhibition to alter thermal responses to LPS. Future systematic studies of the contribution of these other circumventricular organs will probably shed light on other immune signal entry points to the brain and may identify other populations that function in parallel with the NTS–AP to control sickness behaviours.

The NTS is the principal target of the nodose ganglion, which is the site of the cell bodies of afferent vagal neurons, and plays an important role in relaying vagal signals to higher centres^21,31^. The AP is the site of a circumventricular organ and responds to a series of blood-borne signals, in particular those that induce nausea and reduce food intake^22,25,32–34^. This is consistent with our finding that activating several different AP populations can decrease feeding, drinking and locomotion. However, our finding that inhibiting AP neurons does not alter the sickness response is consistent with previous studies showing that blockade of GDF15 signalling by using *Gfral*-knockout mice fails to diminish the anorectic response^35^. The possibility that neurons in the AP may not contribute to the sickness response is also consistent with previous studies showing that inhibiting CGRP neurons in the parabrachial nucleus, a major target of neurons in the AP, has little effect on the anorexia seen during a sickness response^13^. A possible role of ADCYAP1^+^ neurons in NTS raises the possibility that sickness might be mediated by peripheral signals conveyed through vagal and/or spinal afferents that ultimately activate ADCYAP1^+^ neurons in the NTS (and possibly AP). However, this does not exclude the possibility that ADCYAP1^+^ neurons may also be activated by other mechanisms such as direct or indirect interaction with cytokines and humoral immune factors^36^. It is also possible that LPS might interact with Toll receptors on glia or neurons.

LPS activates peripheral and central immune cells through Toll-like receptors, which in turn induces an array of cytokines^9^. It has been shown that multiple immune-derived cytokines, including IL-1β and TNF, are able to elicit sickness behaviours, but it is unclear whether these are directly sensed by the central nervous system, relayed by the peripheral nervous system or induce a set of secondary messengers that convey information about the infectious state^36^. The NTS–AP region was of particular interest because of the ability of this region to directly receive signals from the vagus nerve as well as direct neuronal communication from humoral signals. snRNA-seq experiments of LPS-activated neurons revealed the expression of cytokine receptors in NTS–AP ^ADCYAP1^ (for example, IL-1R1, IL-1R2 and TNFRSF1b) and other cell types (see data from Fig. 4, GSE206144). In addition, glial cells also express cytokine receptors and could also function as an important intermediary^37–39^. Thus, further study of the stimuli activating NTS and AP neurons after LPS injection may provide new insights into the specific mechanisms responsible for neuroimmune crosstalk during infection. Analysing the effect of these mediators on ADCYAP1 neural activity now provides an opportunity to explore the specific signals that are responsible for the ability of LPS to activate sickness behaviours.

We also find that the response to LPS and to ADCYAP1 activation persists well past the time when the stimulus has abated. For example, LPS has a half-life of a few minutes to hours^40^, yet even a modest single dose shows effects for 24 h, and a larger dose elicits effects 72 h later. Although this could be a result of a series of immune events leading to a cascade of cytokine signals, we also find that a single dose of CNO, which has a half-life of about 2 h (ref. ^41^) and thus only transiently activates ADCYAP1^+^ neurons, is also capable of reducing food and water intake and locomotion for 24 h. This protracted effect is consistent with the results of other studies that have shown that inflammatory cytokines such as TNF are able to induce plasticity in neurons. We used *Fos* as a marker for neural activation, but this gene is also an immediate early gene that can also serve as an important marker of transcriptional plasticity^42,43^. Thus, our finding that FOS expression is induced in NTS–AP neurons and that ADCYAP1 neural activation has a protracted effect further suggests that there may be intrinsic plasticity in the NTS–AP and possibly other brain regions in response to LPS^42,43^. This raises the possibility that ADCYAP1^+^ neural activation induces plasticity locally or downstream contributing to the protracted nature of the sickness behaviour response. However, this response must be tuned by other factors eventually limiting its duration as the sickness response carries a considerable metabolic cost, with animals losing nearly 10% of their weight within 24 h (Fig. 1j). Thus, other factors or mechanisms must act to attenuate this response, especially with persistent infections. Indeed, a failure to properly modulate the sickness response could exacerbate the morbidity and mortality of infection and other chronic inflammatory diseases. Elucidating the mechanism that controls the duration of the sickness response will require further studies of the temporal response of ADCYAP1^+^ neurons to LPS and CNO as well as mapping the functional targets of these neurons. Imaging of neurons in the NTS–AP is limited at present by high degrees of movement of the medulla, but head-fixed recordings can be used to monitor neural activity in this region over time, and these studies are underway.

Analyses of the time course of FOS expression revealed a stereotyped temporal response with more than 200 regions being activated at later times, possibly revealing an expanded network of LPS-activated neurons, including downstream regions, that could also contribute to the duration of the response. Optogenetics to activate the nerve terminals of NTS–AP ^ADCYAP1+^ neurons in other regions can now be used to map the functional targets of ADCYAP1^+^ neurons and allow us to determine which projection sites mediate the different elements of the sickness response, including effects on feeding, drinking and locomotion, and possibly other autonomic and metabolic responses. We also find that the response to LPS is not altered by activation of AgRP neurons in the hypothalamus, dysfunction of the leptin axis or starvation, further suggesting that activation of ADCYAP1^+^ neurons suppresses pathways that maintain homeostatic control food intake (Extended Data Fig. 1d). Further studies will be necessary to establish where and how the LPS-activated neural circuits intersect with those that regulate feeding. Overall, studies of the projections of ADCYAP1^+^ neurons and their function may also reveal whether and how different outputs of these neurons mediate different elements of the sickness response.

In summary, we have identified NTS–AP^ ADCYAP1+^ neurons as a fundamental neural substrate for regulation of sickness behaviours. By elucidating a neural circuit responsible for the system-level physiological and behavioural changes induced in response to infection, these results provide an important new component linking infections, inflammation and behaviour. This has important implications for our understanding of how the immune system interacts with the brain.

## Methods

### Mice

Animal experiments were approved by The Rockefeller University Animal Care and Use Committee and were carried out in accordance with the National Institutes of Health guidelines. All experiments were conducted on male mice older than 8 weeks of age housed in a 12-h light/12-h dark cycle at 22 °C and 30–60% humidity. We used the following genotypes of mice: C57BL/6J (wild type; number 000664, The Jackson Laboratory), FOS^2A-iCreER^ (TRAP2) mice (The Jackson Laboratory, stock number 030323), CAG–Sun1/sfGFP (INTACT) mice (The Jackson Laboratory, stock number 021039), VGLUT2–IRES–Cre mice (The Jackson Laboratory, stock no. 028863), VGAT–IRES–Cre mice (The Jackson Laboratory, stock no. 028862), ADCYAP1–2A–Cre mice (The Jackson Laboratory, stock number 030155), PHOX2B–Cre (The Jackson Laboratory, Stock 016223), DBH–Cre (The Jackson Laboratory, stock number 033951), TH–Cre (The Jackson Laboratory, stock number 008601), TAC1–Cre (The Jackson Laboratory, stock number 021877), PDYN–Cre (The Jackson Laboratory, stock number 027958), CCK–Cre (The Jackson Laboratory, stock number 012706), NTS–Cre (The Jackson Laboratory, stock number 017525). For all Cre mouse line experiments, only heterozygous animals were used. Sample sizes were decided based on experiments from similar studies. Animals were randomly assigned to groups when possible.

### Viral vectors

For chemogenetic studies, AAV5-hSyn-DIO-hM3D(Gq)–mCherry (activating), AAV5-hSyn-DIO-hM4Di(Gi)–mCherry (inhibitory) or AAV5-hSyn-DIO–mCherry (control) was used. AAV5 serotype was used as we observed minimal tropism for dorsal motor vagus neurons. All viruses were obtained from Addgene.

### Stereotactic surgery

Animals were anaesthetized using isofluorane anaesthesia (induction 3%, maintenance 1.5–2%). For the NTS–AP region, the following coordinates relative to the bregma were used: anterior–posterior, −7.7 mm to −7.8 mm; medial–lateral, ± 0.1 mm; and dorsal–ventral, −4.8 mm. For chemogenetics, 25–50 nl of virus was injected bilaterally at a rate of 1 nl s^−1^ using a Drummond Scientific Nanoject III Programmable Nanoliter Injector.

### LPS administration

LPS from *Escherichia coli* (O55:B5; Sigma-Aldrich L2880) was resuspended in saline (3 mg ml^−1^) and frozen into single-use aliquots. Further dilutions in saline to the appropriate experimental dose and a standardized volume (about 100 µl per animal) were further generated before each experiment. All doses were delivered with a single i.p. injection.

### Phenotyping measurements and analysis

Animals were phenotyped in custom home-cages consisting of a modified Feeding Experimentation Device 2.0 (refs. ^44,45^) for single-pellet-based food intake measurement, a capacitive lickometer for lick-based water intake measurement and an infrared-compatible camera for movement measurement. Measurements were continuously collected at about 100 Hz for food and water intake and eight frames per second for movement. All data were collected using Bonsai^46^. Mice were housed singly and allowed at least 1 week to habituate to the phenotyping cages before any data were acquired. Data were post hoc analysed using custom MATLAB code to identify pellet withdrawal events and licking events, and with ToxTrac^47^ and MATLAB code for movement. For normalized plots of food intake, water intake and movement, data for each animal were analysed with respect to cumulative measurements of each respective parameter for 24 h for that respective animal without any treatment (baseline behaviour).

### Thermal measurements

Measurements of core body temperature were obtained using an anal probe (Braintree Scientific). BAT temperature measurements were obtained using wireless implantable temperature probes (IPTT-300, Bio Medic Data Systems). Further temperature characterization was visualized with a thermal camera (FLIR Systems) taking at least three images at each respective time point reported.

### AdipoClear whole-brain clearing and imaging

Mice were euthanized by intracardial perfusion with 1× PBS followed by 4% paraformaldehyde. Two saline-treated groups were collected at 0.5 h and 1 h post injection of saline as previous experiments have shown that FOS patterns for saline treatment do not change beyond the first hour after injection. Six LPS-treated groups were collected at 0.5 h, 1 h, 3 h, 6 h, 12 h and 24 h post injection of 0.5 mg kg^−1^ LPS. Brains were carefully dissected and post-fixed in 4% paraformaldehyde for 16 h at 4 °C before proceeding with the AdipoClear protocol as previously described^18^ with the exception of primary and secondary antibody incubations conducted at 37 °C and for 6 days per incubation. Synaptic Systems rabbit c-FOS antibody (number 226003) or a custom guinea pig c-FOS antibody (courtesy of Zuckerman Institute Antibody Core) was used for primary staining at 1:2,500 and 1:25,000 respectively, and a donkey anti-rabbit Alexa Fluor 647 (Thermo Fisher Scientific A-31573) secondary antibody at 1:2,500 was used. Samples were stored in dibenzyl ether at room temperature until imaging. Samples were imaged in a dibenzyl ether reservoir using a light-sheet microscope (Ultramicroscope II, LaVision Biotec) equipped with a 4× objective lens for FOS imaging and 1.3× lens for autofluorescence imaging.

### ClearMap analysis of FOS data

FOS and autofluorescence data were analysed and mapped to the Allen Brain Reference Atlas using the ClearMap pipeline as described previously^17^. The 0.5 h LPS time point was normalized to the 0.5 h saline-treated group, whereas all other LPS time points (1 h–24 h) were normalized to the 1 h saline-treated group.

### TRAP2 labelling for chemogenetics

AAV5-hSyn-DIO-Gq–mCherry (Addgene) or AAV5-hSyn-DIO-Gi–mCherry (Addgene) virus was delivered to TRAP2 mice of >8 weeks of age through stereotactic injection into the NTS–AP region. Mice were given at least 3 weeks for recovery post surgery. Habituation for i.p. injection was carried out daily for 1 week using injections of 100 µl saline. Mice were intraperitoneally injected with 4HT at 20 mg kg^−1^ concurrently with saline or LPS (0.5 mg kg^−1^). Mice were allowed to recover and express DREADDs for at least 3 weeks before animals were injected with 1 mg kg^−1^ CNO or saline (control) to assess behaviour. For inhibition experiments, mice were concurrently injected with LPS during CNO delivery. Exposure to LPS, which can reduce subsequent responses, was controlled between saline- and LPS-labelled groups by injecting saline-labelled animals with LPS at least 3 days after 4HT injection.

### TRAP2 labelling for TRAP2-seq

TRAP2 animals were bred to INTACT (Cre-dependent Sun1–GFP) animals to produce heterozygous animals for both alleles. Animals of about 8 weeks of age were habituated to i.p. injections daily for 1 week using injections of 100 µl of saline before labelling with 4HT and LPS similarly to TRAP2 labelling for chemogenetics.

### Isolation of nuclei for snRNA-seq

Animals were euthanized through transcardial perfusion using a HEPES–sucrose solution (NaCl 110 mM, HEPES 10 mM, glucose 25 mM, sucrose 75 mM, MgCl_2_ 6H_2_O 7.5 mM, KCl 2.5 mM)^48^. Brains were dissected on ice, frozen using LN_2_ and stored at −80 °C until isolation of nuclei. Samples were then processed according to a modified version of a previously described protocol^49^. On the day nuclei were extracted, samples were thawed on ice, resuspended in HD buffer (tricine KOH 10 mM, KCl 25 mM, MgCl_2_ 5 mM, sucrose 250 mM, 0.1% Triton X-100, SuperRNaseIn 0.5 U ml^−1^, RNase Inhibitor 0.5 U ml^−1^), and homogenized using a 1-ml dounce homogenizer. Homogenates were filtered using a 40-μM filter, centrifuged at 600*g* for 10 min and resuspended in nucleus storage buffer (sucrose 166.5 mM, MgCl_2_ 10 mM, Tris buffer pH 8.0 10 mM, SuperRNaseIn 0.05 U ml^−1^, RNase Inhibitor 0.05 U ml^−1^) for staining. Nucleus quality and number were assessed using an automated cell counter (Countess II, Thermo Fisher). Nuclei were stained with combinations of Hoechst 33342 (Thermo Scientific H3570; 1:500 per volume), anti-NeuN Alexa Fluor 647-conjugated antibody (Abcam ab190565) (1:500 per volume) and TotalSeq anti-Nuclear Pore Complex Proteins Hashtag antibody (BioLegend 682205) (0.5 mg per million nuclei) for 30 min at 4 °C. Samples were then washed with 10 ml nucleus storage buffer and centrifuged at 600*g* for 10 min. Nuclei were resuspended in PBS with 2% BSA and RNase inhibitors (SuperRNaseIn 0.5 U ml^−1^, RNase Inhibitor 0.5 U ml^−1^) for subsequent fluorescence-activated cell sorting. Samples were gated on Hoechst to identify nuclei followed by a subsequent gate on high Alexa Fluor 647 expression level for NeuN^+^ nuclei designating neurons.

### snRNA-seq of the NTS–AP

Nuclei were captured and barcoded using 10x Genomics Chromium v3 according to the manufacturer’s protocol. Samples were processed and libraries were prepared by The Rockefeller Genomics Core. Sequencing was performed by Genewiz using Illumina sequencers.

### snRNA-seq analysis

The FASTQ file for each sample was analysed with Cell Ranger version 5.0. The following analysis was based on Orchestrating Single-Cell Analysis from Bioconductor (v1.0.6; https://bioconductor.org/books/release/OSCA/) and vignettes from Seurat (v4.0; https://satijalab.org/seurat/)^50^. The ambient RNA contamination and doublets were estimated by using DropletUtils (https://bioconductor.org/packages/release/bioc/html/DropletUtils.html) and scDblFinder (https://bioconductor.org/packages/release/bioc/html/scDblFinder.html), respectively. After removing ambient RNA, doublets and nuclei with >1% mitochondrial reads, the data from individual samples were integrated using canonical correlation analysis from Seurat using the first 2,000 variable features of each sample. The counting matrices of the samples were then merged and rescaled by using the common variable features of the samples. Finally, a single-cell dataset with 29,329 (features) × 19,918 (cell barcodes) was generated. To optimize the clustering and projection, we tested the principal component analysis up to 40 principal components and evaluated the optimal cutoff by using an elbow plot. In this case, we took the first 25 principal components for clustering and projection with both (UMAP) and *t*-distributed stochastic neighbour embedding. We also tested the resolution of clustering from 0.1 to 1.0. The results were evaluated with clustree (https://github.com/lazappi/clustree). In this dataset, the resolution was set to 1.0, resulting in 44 clusters in this dataset. The clustering and projection information was also applied to generate a customized LOUPE file for further analysis and visualization.

### Chemogenetics for activation or inhibition

AAV5-DIO-Gq (Addgene), AAV5-DIO-Gi (Addgene) or AAV5-DIO–mCherry (Addgene) virus was delivered to respective Cre line mice of >8 weeks of age through stereotactic injection into the NTS–AP region. Mice were allowed to recover and express DREADDs for at least 4 weeks. For activation, animals were injected with 1 mg kg^−1^ of CNO or saline (control). For inhibition, animals were injected with 1 mg kg^−1^ of CNO or saline (control) concurrent with LPS (0.5 mg kg^−1^).

### Immunofluorescence

Animals were transcardially perfused with PBS followed by 4% paraformaldehyde. Brains were dissected and post-fixed for 16–24 h at 4 °C. Brains were sectioned using a vibratome (Leica) to a thickness of 50 µm. Samples were then stained with the following primary antibodies: anti-GFP (Aves Labs GFP-1020) at 1:1,000, anti-mCherry (Rockland 600-401-379) at 1:1,000 or anti-FOS antibody (Synaptic Systems 226 003) at 1:2,000 in PTxwH buffer for 24 h at 4 °C. After four 15-min washes in PTxwH buffer, samples were incubated in Alexa Fluor-conjugated secondary antibodies (Thermo Fisher) at 1:2,000 for 90 min at room temperature, followed by four further 15-min washes before mounting using 4′,6-diamidino-2-phenylindole Fluoromount-G (SouthernBiotech 0100-20 OB010020).

### Statistical analysis

Statistical parameters for individual experiments are reported as sample size (*n* represents the number of animals per group), statistical test used and statistical significance. ANOVA denotes two-way ANOVA. All data and graphs are shown as mean ± s.e.m. unless noted otherwise. Significance is defined as *P* < 0.05, with significance annotations of *P < 0.05, **P < 0.01, ***P < 0.001 and ****P < 0.0001. All statistical analysis was performed using GraphPad Prism, MATLAB, R, Python or ClearMap.

### Reporting summary

Further information on research design is available in the Nature Research Reporting Summary linked to this article.

## Online content

Any methods, additional references, Nature Research reporting summaries, source data, extended data, supplementary information, acknowledgements, peer review information; details of author contributions and competing interests; and statements of data and code availability are available at 10.1038/s41586-022-05161-7.

## Supplementary information

Reporting Summary

## Data Availability

The data that support the findings of this study are available from the corresponding authors upon reasonable request. Requests for reagents should be directed to J.M.F. Raw and processed data have been deposited in the Gene Expression Omnibus and are available under the accession number GSE206144. Source data are provided with this paper.

## Source data

Source Data Fig. 1
Source Data Fig. 2
Source Data Fig. 3
Source Data Fig. 4
Source Data Fig. 5
Source Data Fig. 6
Source Data Extended Data Fig. 1
Source Data Extended Data Fig. 2
Source Data Extended Data Fig. 3
Source Data Extended Data Fig. 4
Source Data Extended Data Fig. 5
Source Data Extended Data Fig. 6
