# Supplementary material for: Brainstem ADCYAP1+ neurons control multiple aspects of sickness behaviour
Source: Nature. 2022 Sep 7;609(7928):761–71. doi: 10.1038/s41586-022-05161-7 (PMC9492535; doi:10.1038/s41586-022-05161-7)
Supplement: Supplementary file 1 — Reporting Summary [file 41586_2022_5161_MOESM1_ESM.pdf]

nature portfolio

Corresponding author(s): Jeffrey Friedman

Last updated by author(s): 06/25/2022

Reporting Summary

Nature Portfolio wishes to improve the reproducibility of the work that we publish. This form provides structure for consistency and transparency in reporting. For further information on Nature Portfolio policies, see our Editorial Policies and the Editorial Policy Checklist.

Statistics

For all statistical analyses, confirm that the following items are present in the figure legend, table legend, main text, or Methods section.

n/a	Confirmed	
☐	☒	The exact sample size (*n*) for each experimental group/condition, given as a discrete number and unit of measurement
☒	☐	A statement on whether measurements were taken from distinct samples or whether the same sample was measured repeatedly
☐	☒	The statistical test(s) used AND whether they are one- or two-sided<br>*Only common tests should be described solely by name; describe more complex techniques in the Methods section.*
☒	☐	A description of all covariates tested
☐	☒	A description of any assumptions or corrections, such as tests of normality and adjustment for multiple comparisons
☐	☒	A full description of the statistical parameters including central tendency (e.g. means) or other basic estimates (e.g. regression coefficient) AND variation (e.g. standard deviation) or associated estimates of uncertainty (e.g. confidence intervals)
☐	☒	For null hypothesis testing, the test statistic (e.g. F, t, r) with confidence intervals, effect sizes, degrees of freedom and P value noted<br>*Give P values as exact values whenever suitable.*
☒	☐	For Bayesian analysis, information on the choice of priors and Markov chain Monte Carlo settings
☒	☐	For hierarchical and complex designs, identification of the appropriate level for tests and full reporting of outcomes
☒	☐	Estimates of effect sizes (e.g. Cohen's *d*, Pearson's *r*), indicating how they were calculated

Our web collection on statistics for biologists contains articles on many of the points above.

Software and code

Policy information about availability of computer code

Data collection	Bonsai 2.4 was used for behavioral data collection, Bioconductor version 1.0.6 (DropletUtils , cDblFinder), Seurat version 4.0, clustree version 0.4.4, BD FACSDiva version 9.0
Data analysis	MATLAB 2020a was used for analysis of behavioral data and statistical analysis where noted using standard functions. Prism 9 was used for plotting and statistical analysis. ClearMap/Python was used for analysis of AdipoClear FOS data and mapping. BioConductor and Seurat 4.0 were used for single nuclei RNA sequencing analysis

For manuscripts utilizing custom algorithms or software that are central to the research but not yet described in published literature, software must be made available to editors and reviewers. We strongly encourage code deposition in a community repository (e.g. GitHub). See the Nature Portfolio guidelines for submitting code & software for further information.

Data

Policy information about availability of data

All manuscripts must include a data availability statement. This statement should provide the following information, where applicable:

- Accession codes, unique identifiers, or web links for publicly available datasets
- A description of any restrictions on data availability
- For clinical datasets or third party data, please ensure that the statement adheres to our policy

The data that support the findings of this study are available from the corresponding authors upon reasonable request. Requests for reagents should be directed to J.F.. Raw and processed data have been deposited in the Gene Expression Omnibus and are available under accession number GSE206144. Source data are provided with this paper.

Field-specific reporting

Please select the one below that is the best fit for your research. If you are not sure, read the appropriate sections before making your selection.

☒ Life sciences ☐ Behavioural & social sciences ☐ Ecological, evolutionary & environmental sciences

For a reference copy of the document with all sections, see nature.com/documents/nr-reporting-summary-flat.pdf

Life sciences study design

All studies must disclose on these points even when the disclosure is negative.

Sample size	No statistical methods were used to calculate sample sizes. Sample sizes were determined based on similar behavioral studies in the field.
Data exclusions	Preeestablished criteria was used for inclusion/exclusion of data from studies. For studies based on stereotactic injection, animals were excluded from data if post-hoc analysis of their injections sites deemed them as not having appropriate injections in the regions of interest.
Replication	Experiments were performed across multiple animal replicates, as denoted in each experiment. All attempts at replication were successful.
Randomization	Mice were randomly assigned to control and experimental groups and littermates were used when possible.
Blinding	Behavioral data was collected and analyzed blind to whether animals were in control or experimental group. Determining whether animal injection sites were valid for inclusion/exclusion from data was performed blind to results for that animal.

Reporting for specific materials, systems and methods

We require information from authors about some types of materials, experimental systems and methods used in many studies. Here, indicate whether each material, system or method listed is relevant to your study. If you are not sure if a list item applies to your research, read the appropriate section before selecting a response.

Materials & experimental systems

n/a	Involved in the study	
☐	☒	Antibodies
☒	☐	Eukaryotic cell lines
☒	☐	Palaeontology and archaeology
☐	☒	Animals and other organisms
☒	☐	Human research participants
☒	☐	Clinical data
☒	☐	Dual use research of concern

Methods

n/a	Involved in the study	
☒	☐	ChIP-seq
☐	☒	Flow cytometry
☒	☐	MRI-based neuroimaging

Antibodies

Antibodies used	1:1000 anti-GFP (Aves Labs GFP-1020); 1:1000 anti-mCherry (Rockland 600-401-379); 1:2000 anti-FOS antibody (Synaptic Systems 226 003); anti NeuN-AlexaFluor647 conjugated antibody (Abcam ab190565) (1:500 per volume); TotalSeq anti-Nuclear Pore Complex Proteins Hashtag antibody (BioLegend 682205)(0.5 mg/million nuclei); 1:2000 donkey anti-rabbit Alexa Fluor 647 (Thermo Fisher Scientific A-31573)
Validation	GFP1020 - PMIDs: 31372393,30850733<br>mCherry - PMIDs: 31257028, 33854415<br>anti-Neun - PMIDs: 33882306, 33013649<br>anti-Nuclear pore complex - PMIDs: 12556490, 11448990<br>Synaptic Systems 226 003 - PMIDs: 31257028

Animals and other organisms

Policy information about studies involving animals; ARRIVE guidelines recommended for reporting animal research

Laboratory animals	Adult male (8-16 weeks) mice from strains: C57BL/6J (wild-type; #000664, The Jackson Laboratory), Fos2A-iCreER (TRAP2) mice (The Jackson Laboratory, Stock #030323), CAG-Sun1/sfGFP (INTACT) mice (The Jackson Laboratory, Stock #021039), Vglut2-ires-Cre mice (The Jackson Laboratory, Stock #028863), Vgat-ires-Cre mice (The Jackson Laboratory, Stock #028862), Adcyap1-2A-Cre mice (The Jackson Laboratory, Stock #030155), Phox2b-Cre (The Jackson Laboratory, Stock 016223), Dbh-Cre (The Jackson Laboratory, Stock #033951), Th-Cre (The Jackson Laboratory, Stock #008601), Tac1-Cre (The Jackson Laboratory, Stock #021877), Pdyn-Cre (The Jackson Laboratory, Stock #027958), CCK-Cre (The Jackson Laboratory, Stock #012706), NTS-cre (The Jackson Laboratory, Stock #017525).

Wild animals	This study did not involve wild animals
Field-collected samples	This study did not involve samples collected in the field
Ethics oversight	Animal experiments were approved by The Rockefeller University Animal Care and Use Committee and were done in accordance with the National Institutes of Health guidelines

Note that full information on the approval of the study protocol must also be provided in the manuscript.

Flow Cytometry

Plots

Confirm that:

- [ ] The axis labels state the marker and fluorochrome used (e.g. CD4-FITC).
- [ ] The axis scales are clearly visible. Include numbers along axes only for bottom left plot of group (a 'group' is an analysis of identical markers).
- [ ] All plots are contour plots with outliers or pseudocolor plots.
- [ ] A numerical value for number of cells or percentage (with statistics) is provided.

Methodology

Sample preparation	*Describe the sample preparation, detailing the biological source of the cells and any tissue processing steps used.*
Instrument	*Identify the instrument used for data collection, specifying make and model number.*
Software	*Describe the software used to collect and analyze the flow cytometry data. For custom code that has been deposited into a community repository, provide accession details.*
Cell population abundance	*Describe the abundance of the relevant cell populations within post-sort fractions, providing details on the purity of the samples and how it was determined.*
Gating strategy	*Describe the gating strategy used for all relevant experiments, specifying the preliminary FSC/SSC gates of the starting cell population, indicating where boundaries between "positive" and "negative" staining cell populations are defined.*

- [ ] Tick this box to confirm that a figure exemplifying the gating strategy is provided in the Supplementary Information.
